# Clinical decision support methods for children and youths with mental health disorders in primary care

**DOI:** 10.1093/fampra/cmac051

**Published:** 2022-06-03

**Authors:** Lennard T van Venrooij, Vlad Rusu, Robert R J M Vermeiren, Roman A Koposov, Norbert Skokauskas, Matty R Crone

**Affiliations:** Department of Research and Education, Academic Center for Child and Youth Psychiatry, Curium-LUMC, Oegstgeest, the Netherlands; Department of Public Health and Primary Care, Leiden University Medical Center (LUMC), Leiden, the Netherlands; Department of Research and Education, Academic Center for Child and Youth Psychiatry, Curium-LUMC, Oegstgeest, the Netherlands; Department of Research and Education, Academic Center for Child and Youth Psychiatry, Curium-LUMC, Oegstgeest, the Netherlands; Youz, Parnassia Psychiatric Institute, the Hague, the Netherlands; Regional Centre for Child and Youth Mental Health and Child Welfare, Northern Norway, UiT, The Arctic University of Norway, Tromsø, Norway; Sechenov First Moscow State Medical University, Moscow, Russia; Regional Centre for Child and Youth Mental Health and Child Welfare, IPH, Faculty of Medicine and Health Sciences, NTNU, Trondheim, Norway; Department of Public Health and Primary Care, Leiden University Medical Center (LUMC), Leiden, the Netherlands

**Keywords:** access to care, adolescent, children and youths, family health, mental health, primary care

## Abstract

**Background:**

Mental health disorders among children and youths are common and often have negative consequences for children, youths, and families if unrecognized and untreated. With the goal of early recognition, primary care physicians (PCPs) play a significant role in the detection and referral of mental disorders. However, PCPs report several barriers related to confidence, knowledge, and interdisciplinary collaboration. Therefore, initiatives have been taken to assist PCPs in their clinical decision-making through clinical decision support methods (CDSMs).

**Objectives:**

This review aimed to identify CDSMs in the literature and describe their functionalities and quality.

**Methods:**

In this review, a search strategy was performed to access all available studies in PubMed, PsychINFO, Embase, Web of Science, and COCHRANE using keywords. Studies that involved CDSMs for PCP clinical decision-making regarding psychosocial or psychiatric problems among children and youths (0–24 years old) were included. The search was conducted according to PRISMA-Protocols.

**Results:**

Of 1,294 studies identified, 25 were eligible for inclusion and varied in quality. Eighteen CDSMs were described. Fourteen studies described computer-based methods with decision support, focusing on self-help, probable diagnosis, and treatment suggestions. Nine studies described telecommunication methods, which offered support through interdisciplinary (video) calls. Two studies described CDSMs with a combination of components related to the two CDSM categories.

**Conclusion:**

Easy-to-use CDSMs of good quality are valuable for advising PCPs on the detection and referral of children and youths with mental health disorders. However, valid multicentre research on a combination of computer-based methods and telecommunication is still needed.

**Key messages**

- Mental health disorders in young people are common and have negative consequences.
- Primary care providers (PCPs) play a crucial role in the detection and management.
- Clinical decision support methods (CDSMs) can assist PCP decision-making.
- Computer-based CDSMs focus on self-help, diagnosis, and treatment suggestions.
- Telecommunication methods offer support using interdisciplinary (video) calls.
- Future efforts should aim at a combination of both identified categories of CDSMs.

## Background

Mental health disorders among children and youths are common, as an estimated 10–20% of them experience mental health difficulties.^1,2^ All too often, mental health disorders remain underdiagnosed and undertreated.^3^ A continued disparity exists between the increasing demands for paediatric mental health services and the limited supply of these services, particularly because of a shortage of child and adolescent psychologists and psychiatrists.^4–7^ To prevent negative long-term consequences for families and economic burdens for communities, accurate and timely detection of mental health disorders and appropriate referrals to youth mental health care are essential.^8,9^ One in four 7–12 year olds and four in ten 13–16 year olds who attend primary care have some sort of mental health problem.^10^ Therefore, primary care providers (PCPs) play an important role in the detection of mental health disorders and referrals to specialist services.^10^ In most Western countries, general practitioners (GPs) and paediatricians are examples of PCPs.^11^ Despite their crucial role, PCPs report a profound lack of communication skills with children and adolescents and a lack of confidence and knowledge about mental health difficulties, which negatively affect their clinical decision-making.^8,9,12^ Furthermore, collaborative care between PCPs and specialist child and youth mental health care providers is not satisfactory in terms of interdisciplinary communication and logistic procedures, for example, the quality of provided patient-specific information in referral letters.^9^

To improve the detection of mental health disorders and referral efficacy, various approaches have been developed to support PCPs in their clinical decision-making, including clinical decision support methods (CDSMs).^8^ Currently, no universal definition of CDSM exists. Therefore, this study uses the CDSM definition by Sim et al.: “methods that are designed to be a direct aid to clinical decision-making, in which the characteristics of an individual patient are matched to a (computerized) clinical knowledge base and patient-specific assessments or recommendations are then presented to the clinician or the patient for a decision”.^13^ Overall, CDSMs are aimed at the clinician analysing the current condition of the patient and providing support regarding treatment or referral, whereas decision aids are aimed at patients, offering choices regarding medical treatment. However, similar to decision aids, some CDSMs may encourage patients to participate actively in healthcare decisions.^14^

There are remarkable differences between non-computer-based and computer-based CDSMs, although previous research has mainly focused on computer-based CDSMs. One systematic review concluded that there is a need for readily available systems that promote evidence-based practices. These systems should consider regional variations in practice. They should leverage data reuse to generate predictions regarding treatment outcomes and address a broader cluster of clinical disorders. Furthermore, these systems should target primary care practices with limited knowledge and skills regarding child and adolescent psychiatry.^8^ Research on non-computer-based CDSMs, such as child psychiatry access programmes, recommended more investigations on the broad impact of these programmes on, for example, patients, families, or health systems instead of more descriptive evaluations focusing on programme usage and provider satisfaction.^15^

The present systematic review aimed to identify CDSMs for primary care that support clinical decision-making regarding children and youths with mental health disorders. To this end, a distinction was made between non-computer-based and computer-based CDSMs. The objective of this review was to describe the functionalities of CDSMs and their capability to provide diagnostic and referral support. Furthermore, we assessed the content of CDSMs and the quality of the underlying studies.

## Methods

### Search strategy

To identify all available studies, published between 2009 and 2021, that have described CDSMs for mental health disorders in children and youths in primary care, PubMed, PsychINFO, Embase, Web of Science, and COCHRANE were searched in August 2021. A combination of the following keywords was used in the search strategy: “efficacy”, “clinical decision-making”, “support”, “triage methods”, “general practitioner”, “psychiatry”, “mental health disorders”, “child”, “adolescent”, “primary care”, and “secondary care”. By consensus, LV, VR, and an information expert specifically selected each keyword and potential synonym. Questions related to keyword selection were discussed with MC. The detailed search strategy is described in the Supplementary Material.

### Inclusion and exclusion criteria

Inclusion and exclusion criteria were determined prior to the keyword search. Peer-reviewed studies that described CDSMs for mental health disorders among children and youths (0–24 years) were included. By “CDSM”, the authors mean a method (a procedure, e.g., digital support) that assists PCPs in assessing children and youth with mental health symptoms and in deciding the need for referrals to specialized mental health care, preventive care, or primary care support. The search was limited to publications in English and Dutch. Studies were excluded if the recruited participants were all aged 25 years or older and if the methods used fully consisted of a dichotomous screening instrument.^16^

### Selection procedure

**Fig. 1.**
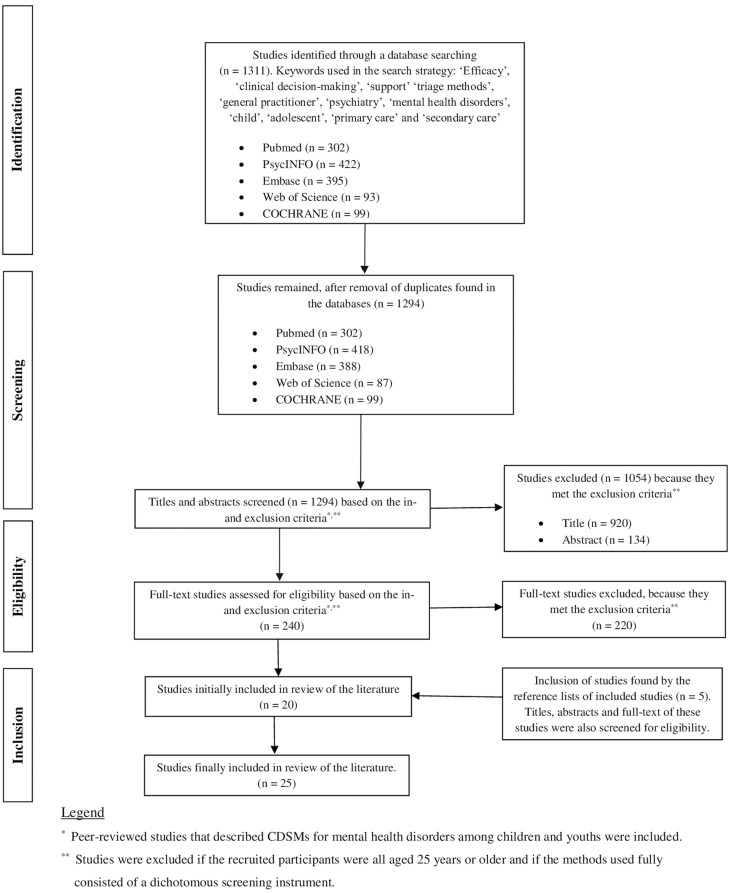
Flowchart regarding the selection of literature, 1,294 studies published in 2009–2021.

Titles and abstracts from all identified studies were reviewed by LV and VR based on inclusion eligibility. Based on the inclusion and exclusion criteria described above, titles and abstracts were categorized into “to include”, “questionable”, and “to exclude”. Questions raised with regard to studies labelled as “questionable” were discussed with MC prior to being labelled as “to include” or “to exclude”. For example, there was a discussion about whether some studies fulfilled the criteria for CDSM; that is, the method was more focused on the assessment of mental health problems instead of supporting the decision regarding follow-up care. Full-text studies labelled as “to include” were read by LV and VR while extracting information as described below. Figure 1 describes a detailed flowchart concerning the inclusion and exclusion process. The systematic review was conducted in accordance with the Preferred Reporting Items for Systematic Reviews and Meta-Analyses Protocols (PRISMA-Protocols).^17^

### Variables extracted

Based on the study methodology as mentioned in previous research, categories of information to be extracted were assembled by LV, VR, and MC by reaching a consensus.^8^ No efforts were made to synthesize outcomes because of the variability of the results presented by the studies reviewed. Extracted variables regarding the content of the CDSM were (abbreviated) method name, target population, targeted professionals, goal, content and organization of the method, and phase(s) of clinical decision-making that are supported by the CDSM: diagnosis, assessment of severity, and management.^18^ We also extracted variables regarding study design: objectives, methods, outcomes on provider (e.g., user satisfaction), patient level (e.g., referral efficacy), measurement moments and study duration, name of intervention, control group characteristics, target group characteristics, number of study participants, gender ratios, and mean ages of patients, as well as results at the provider and patient levels. The quality of the included studies was appraised by LV and VR using the Crowe Critical Appraisal Tool (CCAT), which helps in rating the studies included in a systematic review.^19^ CCAT helps readers with different levels and types of knowledge to reach similar conclusions about a research paper. The CCAT is one of the few instruments that has undergone both reliability and validity evaluations and is able to appraise different research study designs. The tool has been used broadly in previous research.^19–22^ The CCAT consists of a 22-item form divided into eight categories—preliminaries, introduction, design, sampling, data collection, ethical matters, results, and discussion of a study—which are scored by readers on a 6-point scale from 0 to 5. Each study is assigned a score on these categories, and the total score is obtained as a sum of all category scores (ranging from 0 to 40).^23^ LV scored all included studies before reaching agreement on scores with VR.

## Results

After the removal of duplicates, the search strategy yielded 1,294 studies across different databases. Some studies were excluded based on the eligibility of titles and abstracts. Another set of studies was excluded because they met the exclusion criteria based on reading their full texts. Lastly, we added studies found in the reference lists of some of the included studies. In total, 25 studies describing 18 different CDSMs were included in the review (Fig. 1). All studies were written in English or Dutch.

### Computer-based decision support methods

Of all identified CDSMs, more than half were computer-based decision support methods (CBDSMs) directed at patients aged 0–75 years old. CBDSMs provided electronic support on (clinical) reasoning for patients and providers.^24–37^ For patients, these methods provide tools for assessing (future) symptom severity^34–37^ and consecutive self-management of their mental health.^33^ The methods were also used to integrate service users’ and practitioners’ expertise about mental health to allow shared decision-making.^24^ Providers were given probability diagnoses following evidence-based algorithms based on routine data^27^ and surveys.^28,29,31,34–37^ Furthermore, some methods offered providers treatment suggestions,^24,25^ such as medication management^26,32^ and referral support.^25,35,36^ To achieve this output from the system, specific patient- and provider-related input was necessary. Patient-related input included vital signs and laboratory test results (e.g., body mass index, systolic and diastolic blood pressures, blood lipids, glucose metabolism), as well as questions on a variety of mental health areas (e.g., symptoms, side effects, treatment preferences, adherence, and response).^24,32,34–37^ Non-medical information, such as social life, finances, and school performance, was also retrieved in some methods.^24,28–31^ Provider-related input comprised information in the child’s electronic health record,^34^ health risk questions based on this information, and screening questions following a decision tree.^27–31^ Most computer-based methods focused on a variety of diagnoses.^24,27–29,31,35–37^ However, some focused on one specific disorder or symptom, such as autism spectrum disorder,^25,30,34^ attention-deficit/hyperactivity disorder (ADHD),^26^ and psychosis.^33^ More than half of the CBDSMs supported all phases of clinical decision-making, that is, support of diagnostics, assessment of severity, and management.^25,26,28–31,34–37^ Other methods supported two phases, that is diagnostics and assessment of severity^24,33^ or diagnostics and management^27,32^ (Supplementary Table 1).

Observational,^24,25,27–29,37^ comparative,^26,29,32–36^ or validation study designs^30,31^ were used to study the implementation (including clinical and cost-effectiveness) or the validation of the CBDSMs. The observational studies found that the CBDSMs were generally appreciated by both patients and care providers, for example, regarding a shared understanding of mental health risks, which facilitated implementation into primary practice.^24,27,37^ However, barriers related to workflow were also reported, such as challenging and confusing access to the method, hardware- and software-related difficulties, the need for duplication during the transition from paper to the electronic system, and issues regarding computer literacy.^24,25,27^ The comparative studies showed a reduction in psychological distress compared to usual care^35,36^ and an increase in the rate of diagnostic assessments compared to (community) control samples, which resulted in, for example, more prescriptions and visits.^26,28,32,34^ Furthermore, these studies showed a higher quality of care with respect to ADHD diagnosis.^26^ The studies reported fewer or no side effects^35,36^ and a reduced weight gain when patients used medication.^32^ In one study, the use of the CDSM led to an increased PCP understanding of patient mental health compared to an attention-comparison group, in which daily activities were monitored without monitoring mood and stress.^33^ All validation studies were directed at the Development and Well-Being Assessment (DAWBA). This method showed good test capabilities when compared between groups of low to high risk of autism spectrum disorder or eating disorders, with high sensitivity (88–100%), specificity (85–94%), positive predictive (82–88%), and negative predictive values (90–100%).^30,31^ (Supplementary Table 2). The average study quality of the CBDSMs was three stars (total score of 30.61), according to the CCAT.^24–37^ Lower total scores were attributed to poor description of design and sample of the study, whereas higher scores were attributed to a clear description of the data, as well as results and discussion sections^24–37^ (Table 1).

**Table 1.** Quality appraisal scores of 25 included studies (published 2009–2021), using the Crowe Critical Appraisal Tool (CCAT)

	Total (max = 40)	Score^a^	Prelim	Intro	Design	Sample	Data	Ethics	Results	Discussion
Buckingham (2015)	**★★**	24	4.75	5	2.75	3.33	0.83	2	2.25	3.17
Bauer (2015)	**★★★**	28.67	5	5	3.5	3.17	2.5	2	3.5	4
Downs (2019)	**★★★★**	33.38	5	5	3.25	5	3.97	3.5	4.5	3.16
Carrol (2013)	**★★★**	30.40	5	5	3.75	2.83	3.83	2.5	3.5	4
Fortney (2010)	**★**	20.49	4.5	5	2.5	1.17	2.17	1	1.32	2.83
Goodman (2000)	**★★★**	29.83	5	2.5	4.25	4.67	4	3	3.25	3.17
Ford (2013)	**★★★★**	35.17	5	5	4.75	3.83	4.67	3	4.25	4.67
McEwen (2016)	**★★★★**	34.42	4.75	5	4.5	4.67	4	3	3.5	5
Moya (2005)	**★★★**	32.33	3.75	5	4.5	5	3.33	2	3.75	5
Robinson (2018)	**★★★★**	32.83	4.75	5	4.5	4	3.33	2.5	3.75	5
Reid (2013)	**★★★★**	35.08	5	5	4.5	4.83	3.33	3.5	4.25	3.83
Fletcher (2019 and 2021)^b^	**★★★**	32.23	5	5	2.69	4.91	3.89	3.75	3	3.99
Parker (2020)	**★★★**	29.15	4.75	5	2.61	3.66	3.14	3.5	3	3.49
Kaye (2017)	**★**	22.68	5	5	2.76	3.33	2.83	2	3.75	3.5
Gadomski (2014)	**★★**	25.96	4	5	2.63	3	3.33	1	3.5	3.5
Kerker (2015)	**★**	21.22	3.50	3	2.95	2.91	1.92	1.11	2.19	3.64
Yellowlees (2008)	**★★★**	29.67	4.75	5	3.42	3.5	2.5	2.5	3	5
Epstein (2007)	**★★★**	32	4.75	5	4.25	3.83	2.83	3	3.75	4.5
Williams (2006)	**★**	21.33	4.75	5	2.58	2.5	1.17	1	2	3.33
Jacob (2012)	**★★**	25.87	4.75	5	2.37	2.17	2.83	2.5	2.75	3.5
Walter (2019)	**★★★**	29.23	4.75	5	2.85	4.15	2.98	3	2.5	4
Malas (2019)	**★★**	27.8	4.75	5	3.09	3.49	2.81	2.5	2.5	3.66
Thompson (2019)	**★**	22.3	3.75	5	2.5	2.32	2.15	1.5	2.25	2.83
Campbell (2021)	**★★**	27.21	4.75	5	3.25	2.32	2.15	2.5	2.75	4.49

Max = maximum, Prelim = preliminaries; Intro = introduction.

One star: more than 1 SD below average; two stars: between 1 SD below average and average; three stars: between average and 1 SD above average; four stars: more than 1 SD above average.

Because Fletcher et al.^35^ consist of a protocol that is expanded on in Fletcher et al.,^36^ displayed scores are mean scores of the two studies. For the categories “Results” and “Discussion”, scores for Fletcher et al.^36^ are mentioned.

### Telecommunication methods

Less than half of the identified CDSMs were telecommunication methods targeted at 0–21 year olds. The telecommunication methods consisted of a practice in which PCPs are advised on mental health management through (video) conferences between psychiatrists and patients.^38–46^ These methods offered patients psychoeducation on medication,^45^ illness and diagnostic issues, exercise and lifestyle issues,^43^ and providers recommendations on referral.^39,46^ Some telecommunication methods also offered (peer) training for PCPs as part of the method,^38–40,45,46^ face-to-face assessments for patients if necessary,^39,43^ and strategies for practice transformation to integrate the telecommunication method.^45^ All but one method^42^ focused on multiple mental disorders at once.^38–41,43–46^ In one telecommunication method, there was no contact between psychiatrists and patients, but physicians received advice from psychiatrists on starting dosages of medication based on effect rating scales.^42^ Almost all telecommunication methods supported all phases of clinical decision-making^38–41,43–46^; one method supported two phases, that is, assessment of severity and management^42^ (Supplementary Table 1).

To investigate the telecommunication methods, almost all the studies used an observational design.^38,39,43–46^ One study used a comparative design.^42^ The studies showed behavioural improvement of the child compared with a sample of children not participating in the CDSM,^42^ provider’s and patient’s satisfaction with the method,^44^ PCPs’ knowledge and confidence regarding mental health disorders,^38,39,43,46^ and collaborative treatment between PCP and family^39^ after implementation of the method. Furthermore, the studies reported alleviation of the gap between youth needing quality behavioural health services and those receiving them,^45^ improved mental health in a convenience sample over time,^41^ more psychotropic medication prescriptions compared to a group of PCPs not receiving training for the CDSM,^40^ and increased psychotherapy, medical behavioural health visits, and guideline congruent medications prescriptions^45^ (Supplementary Table 2). The average CCAT score for the telecommunication methods was two stars (total score of 26.20), with lower scores mainly attributed to description of design and used data, and higher scores attributed to description of results and discussion^38–46^ (Table 1).

### Combination of CDSMs

Two identified CDSMs were CDSMs consisting of a combination of computer-based decision support- and telecommunication method-related components. These CDSMs were directed at patients between 16 months of age and patients older than 75 years.^47,48^ One CDSM started with an algorithm in the patient’s electronic health record, which decided whether the patient health questionnaires were completed.^47^ If the questionnaires indicated that the patient needed to be referred based on depression symptoms, there was an option for the PCP to have contact with a child and youth mental health care provider on medication prescriptions.^47^ The other CDSM comprised a screening instrument via the patient’s electronic health record, with the possibility of referring the patient to a multidisciplinary team for autism evaluation as part of the method.^48^ Both CDSMs were directed at one specific disorder.^47,48^ The CDSM described by Thompson et al. supported all phases of clinical decision-making.^47^ The CDSM described by Campbell et al. supported two phases: diagnostics and management.^48^ Thompson et al.’s study used a comparative design with which the effectiveness of screening, referrals, and treatment uptake was measured via analysis of electronic health record data and screening of patients using the Patient Health Questionnaire-2 and -9.^47^ The study by Campbell et al. consisted of a comparative design that implemented process changes in intervention clinics.^48^ Comparisons were made between these intervention clinics and community clinics (which only received automatic reminders as part of the process changes), as well as between phases of change.^48^ Both studies showed an increase in screening and referral rates.^47,48^ The average quality of Thompson et al. and Campbell et al. was one and two stars (total score of 24.76), respectively, with lower scores attributed to poor descriptions of ethics and higher scores attributed to well-described introduction sections^47,48^ (Table 1).

## Discussion

The present literature review aimed to provide a description of the functionalities of CDSMs and their capability to provide diagnostic support and support for management or referral by primary care practitioners (PCP). Furthermore, we examined the content of CDSMs and quality of underlying studies. This review yielded 25 studies describing 18 CDSMs used in primary care.

The majority of the CDSMs were CBDSMs, which provide electronic support for clinical reasoning following an algorithm. These CDSMs assist patients by offering tools for assessing the severity of (future) symptoms and consecutive self-management of their mental health. Moreover, they assist PCPs by offering probability diagnoses and suggestions for further management or referrals. Some functionalities of this category of CDSMs include monitoring tools,^33,37^ screening forms,^25,26,34,37^ a patient registry, a patient encounter scheduler, trial management,^27^ and (self-)assessment instruments^24,27,35,36^ with structured or open-ended questions.^28–31^ The CBDSMs are directed towards mental health disorders and provide PCPs with advice on diagnosis based on data collected before the consultation.

Less than half of the identified CDSMs were telecommunication methods. Through video conferences between psychiatrists and patients, these methods offer patients psychoeducation on multiple mental health topics. Additionally, these methods advise PCPs on mental health management or referrals. Contrary to CBDSMs, telecommunication methods are used to generate advice on diagnosis and referral based on concerns of the PCP during the consultation. Their functionalities comprise education for PCPs to improve detection of mental health disorders,^38–40,42,45^ referral support by phone, e-mail and/or video,^41,42,44–46^ and face-to-face evaluations with patients if necessary.^38–40,43,46^ We found two CDSMs that consisted of a combination of CBDSM- and telecommunication method-related components.^47,48^

There are several pros and cons of the identified CDSMs with regard to their usability in the primary care process as well as their relevance for clinical practice. CBDSMs provide the PCP with more information about possible mental health disorders based on electronic health records^34^ and, if applicable, a previous consultation, information that can be used to structure the next consultation with the child.^32^ For some CBDSMs, this notice is based on data from large studies.^28^ Moreover, children and their parents can have the opportunity to prepare for the consultation, because the CBDSMs stimulate them to think about relevant medical information that may also be discussed with their PCP.^32^ Another advantage is that no other care providers are involved in using the CDSM, except for the PCP.^25^ Therefore, the invested time and costs are limited. There are also disadvantages. First, CBDSMs should not be used in urgent situations because input from children and their parents may be quite time-consuming.^30^ Second, for some patients, computer-based decision support may be difficult to use due to their mental status.^32^ Third, a set of questions received beforehand may give too much direction to the consultation, which may impede children and their parents from talking about one set of problems more than others.^35,36^

An advantage of telecommunication methods over CBDSMs is their usability during consultation with the child. Therefore, information gathered during the conversation can be used directly for the telecommunication method.^41^ Furthermore, telecommunication methods provide room to take the context of the child and its problem into account while generating advice on diagnosis and referral, information that might be missed when using predetermined questions.^44^ A disadvantage of telecommunication methods is that their usage requires time investment from both PCPs and mental health care providers, which also makes them more costly compared to the one-off purchase of CBDSMs.^39^ CDSMs that consist of CBDSM- and telecommunication method-related components may have a combination of the abovementioned advantages and disadvantages.^47,48^

CBDSMs were directed at 0–75 year olds, telecommunication methods at 0–21 year olds, and a combination of these CDSMs at 16 months old, as well as patients older than 75 years. Since this is a broad age range, it should be noted that the applicability of individual CDSMs differs by age category. For example, younger children should be assisted by their parent and/or caregiver while providing information for a CDSM. By contrast, adolescents may be capable of providing information without any help, depending on their age and capability of self-determination.^28–31^ Therefore, PCPs should be aware of national care regulations with regard to the self-determination of young people.^49^

The quality of the underlying studies of CDSMs was variable. Compared to studies describing telecommunication methods, studies describing CBDSMs had a higher quality, that is, with regard to the description of the data. The aims of the studies describing CBDSMs were to describe the functional capabilities of the CDSM,^27^ to validate the CDSM,^28,30,31^ to describe PCP user satisfaction regarding the CDSM,^24,33,37^ to compare care with the CDSM and care without the CDSM with respect to screening rates^34^ and cost-effectiveness.^35,36^ Furthermore, these studies assessed the impact of the CDSM on the patient’s view of their own life and health^24,32^ and explored the effect of using a CDSM on PCP’s knowledge, beliefs, and self-reported practice regarding mental health disorders.^25^ The aims of studies on telecommunication methods included a description of the impact of CDSMs on care (e.g., medication prescriptions, treatment plans)^33,40,42^ and costs,^45^ effectiveness of detection of mental health disorders,^39^ PCP-reported satisfaction with the CDSM, and PCP’s knowledge and confidence regarding mental health disorders.^38,39,44–46^ It is notable that almost all the studies on telecommunication had an observational study design, implying a need for more comparative research designs.^38,39,43–46^ Studies describing CDSMs consisting of a combination of both CDSM types were of low average quality. These studies aimed to analyse the effectiveness of screening, referrals, and treatment uptake of the CDSM, as well as to assess quality improvement related to screening and referrals while implementing process changes.^47,48^

There were a few studies with outcomes specifically directed at ensuring accurate and timely detection of mental health disorders and appropriate referral, mentioned earlier as essential factors for preventing the long-term consequences of mental health disorders in children and youths.^8,9^ Two studies showed an increased rate of diagnostic assessments,^26,47^ while other studies reported more medication visits and prescriptions.^32,40,42,45^ These findings raise discussion about the possible overdiagnosis and overtreatment of mental health disorders due to the usage of CDSMs. Earlier research has confirmed overdiagnosis and overtreatment in children and youths with ADHD.^50^ However, improved detection of these mental disorders may counteract the underdiagnosis and undertreatment that also exists in this population.

### Strengths and limitations

This study has several limitations. First, it was difficult to compare the different studies due to differences in quality, study designs, and outcome measures. Second, some identified CDSMs were directed at a broad age range, including those of 25 years and older, and had generic output (e.g., self-reported medication visits and vital signs). Therefore, it was not always clear how these CDSMs could be beneficial for children and youths specifically. Third, in some studies, it was unclear whether they included also children and youths. However, these studies were included because it was plausible studied CDSMs were directed at adults, children, and youths. Fourth, most studies originated from the United States of America (USA),^25,26,32–34,38,40,41,43,45–48^ the United Kingdom (UK),^24,28–31^ and Australia,^35–37^ which indicates that region-specific healthcare regulations must be taken into account while interpreting the review results. The health systems of the USA, the UK, and Australia are similar in many ways. In these countries, GPs or primary care paediatricians can be approached for first-contact medical care. However, there are also notable differences, such as the “gatekeeper” role for GPs in the UK and Australia.^51–53^ Furthermore, in the USA, access to mental health care can be inadequate, with more than 5,000 mental health professionals in shortage areas, mostly situated in rural areas.^54^ The aforementioned factors influence which CDSMs are suitable for a particular general practice setting. For example, computerized CDSMs might be more suitable if a GP is the only point of entry for care by a specialist, while telecommunication methods might be more appropriate in regions with a shortage of and longer travelling distance to mental health professionals.

This study also has strengths. First, to include relevant studies, the authors used a priori inclusion and exclusion criteria. Second, to minimize errors in the selection and reading process, there were two searchers and readers of studies. Third, this study provides an overview of different types of CDSMs, which may be useful for PCPs with tight schedules, such as GPs. To the best of our knowledge, no current scientific literature provides such an overview.

## Conclusion

To assist PCPs in early detection and management of mental health disorders among children and youths, easy-to-use CDSMs of good quality are needed which can provide advice on management or referral.^8,9,12^ Based on the current review, methods consisting of a combination of CBDSMs and telecommunication methods are advised. While this advice applies to healthcare systems in which there are sufficient resources and care providers, it does not apply to healthcare systems in which there are shortages and where choices have to be made regarding care provision; that is, where CDSMs may be used as an aid for triage. In these systems, clinical assessments of experts in the context of telecommunication methods may be restricted to “severe” cases, as graded by an electronic system as part of a CBDSM. Electronic systems may be used by PCPs in “mild” and “moderate” cases without further clinical assessment by an expert in secondary mental health care. As for future research, we suggest more comparative multicentre studies (e.g., with a prospective cohort design) on a combination of CBDSMs with telecommunication methods in different health systems and different degrees of problem severity. These combined methods may consist of existing or newly researched CDSMs. Identified CDSMs that support multiple phases of clinical decision-making should have priority in future efforts.

## Supplementary Material

cmac051_suppl_Supplementary_Material

cmac051_suppl_Supplementary_Checklist

cmac051_suppl_Supplementary_Table_1

cmac051_suppl_Supplementary_Table_2

## Data Availability

The data underlying this article are available in the article and its online supplementary material.
