# Correction to: Clinical decision support methods for children and youths with mental health disorders in primary care

**DOI:** 10.1093/fampra/cmac088

**Published:** 2022-07-29

This is a correction to: Lennard T van Venrooij, Vlad Rusu, Robert R J M Vermeiren, Roman A Koposov, Norbert Skokauskas, Matty R Crone, Clinical decision support methods for children and youths with mental health disorders in primary care, Family Practice, 2022, cmac051, https://doi.org/10.1093/fampra/cmac051

In the originally published version of this manuscript, the name of author Vlad Rusu was erroneously given as Vlad R. Rusu.

This error has been corrected.

