# Supplementary material for: Clinical decision support methods for children and youths with mental health disorders in primary care
Source: Fam Pract. 2022 Jun 3;39(6):1135–43. doi: 10.1093/fampra/cmac051 (PMC9680662; doi:10.1093/fampra/cmac051)
Supplement: cmac051_suppl_Supplementary_Material [file cmac051_suppl_supplementary_material.docx]

**Supplementary material: detailed search strategy**Pubmed (<http://www.ncbi.nlm.nih.gov/pubmed?otool=leiden>)
((("Trust"[Mesh] OR "trust"[tw] OR trust*[tw] OR "confidence"[tw] OR "confident"[tw] OR "referral planning"[tw] OR "referral plans"[tw] OR "treatment plan"[tw] OR "treatment plans"[tw] OR "therapy plan"[tw] OR "therapy plans"[tw] OR "plan of treatment"[tw] OR "care plan"[tw] OR "care plans"[tw] OR "plan of care"[tw] OR "treatment path"[tw] OR "treatment paths"[tw] OR "therapy pathway"[tw] OR "treatment pathway"[tw] OR "treatment pathways"[tw] OR "care pathway"[tw] OR "care pathways"[tw] OR "physician satisfaction"[tw] OR "physicians satisfaction"[tw] OR "physician's satisfaction"[tw] OR "practitioner satisfaction"[tw] OR "gp satisfaction"[tw] OR "gps satisfaction"[tw] OR "doctor satisfaction"[tw] OR "doctors satisfaction"[tw] OR "doctor's satisfaction"[tw] OR "satisfied physicians"[tw] OR "satisfied gps"[tw] OR ("Personal Satisfaction"[mesh] AND "Physicians"[mesh]) OR "Practice Management"[Mesh] OR "practice management"[tw] OR "office management"[tw] OR "Practice Patterns, Physicians'"[majr] OR "Early Diagnosis"[majr] OR "early detection"[ti] OR "Randomized Controlled Trial"[ptyp] OR "Validation Studies"[ptyp] OR "Follow-Up Studies"[mesh] OR "treatment outcome"[tw] OR "Treatment Outcome"[mesh]) **AND ("Child"[mesh] OR "child"[tw] OR "children"[tw] OR "childhood"[tw] OR "child*"[tw] OR "Adolescent"[mesh] OR "adolescent"[tw] OR "adolescents"[tw] OR "adolesence"[tw] OR "adolescen*"[tw] OR "girl"[tw] OR "girls"[tw] OR "boy"[tw] OR "boys"[tw] OR "youths"[tw] OR "youth"[tw] OR "teen"[tw] OR "teens"[tw] OR "teenager"[tw] OR "teenagers"[tw]) AND ("Triage"[majr] OR "triage"[ti] OR "Referral and Consultation"[majr] OR "Referral"[ti] OR "referrals"[ti] OR "DAWBA"[ti] OR "Diagnostic And Well-Being Assessment"[ti] OR "Diagnostic And Wellbeing Assessment"[ti] OR "development and well being assessment"[ti] OR "development and wellbeing assessment"[ti] OR "well being assessment"[ti] OR "wellbeing assessment"[ti] OR "wellbeing assessments"[ti] OR** "Clinical decision support systems"[ti] OR "Clinical decision support system"[ti] OR "Decision Support Systems, Clinical"[majr]**) AND ("primary health care"[mesh] OR "general practitioners"[mesh] OR "family practice"[mesh] OR "physicians, family"[mesh] OR "nurse practitioners"[mesh] OR "Primary Health Care"[tw] OR "Primary Care"[tw] OR "General Practitioners"[tw] OR "Family Medicine"[tw] OR "Family Physicians"[tw] OR "General Practitioner"[tw] OR "Family Physician"[tw] OR "family doctor"[tw] OR "family doctors"[tw] OR "GP"[tw] OR "GPs"[tw] OR "General Practice"[tw] OR "family practice"[tw] OR "family practitioners"[tw] OR "family practitioner"[tw] OR "family practice"[tw] OR "nurse practitioners"[tw] OR "nurse practitioner"[tw] OR "mental health nurse practitioner"[tw] OR "mental health nurse practitioners"[tw] OR "primary mental health worker"[tw] OR "primary mental health workers"[tw] OR "first line"[tw] OR "firstline"[tw] OR "community"[tw]) AND ("community psychiatry"[mesh] OR "community mental health services"[mesh] OR "Community Psychiatry"[tw] OR "Community Mental Health"[tw] OR "Assertive Community Treatment"[tw] OR "Community Mental Health Services"[tw] OR "Community Counseling"[tw] OR "Community Psychology"[tw] OR "mental health"[mesh] OR "Mental Disorders"[mesh] OR "Mental Disorders"[tw] OR "Adjustment Disorders"[tw] OR "Affective Disorders"[tw] OR "Alexithymia"[tw] OR "Anxiety Disorders"[tw] OR "Autism Spectrum Disorders"[tw] OR "Chronic Mental Illness"[tw] OR "Dementia"[tw] OR "Dissociative Disorders"[tw] OR "Eating Disorders"[tw] OR "Elective Mutism"[tw] OR "Factitious Disorders"[tw] OR "Gender Identity Disorder"[tw] OR "Hoarding Disorder"[tw] OR "Hysteria"[tw] OR "Impulse Control Disorders"[tw] OR "Koro"[tw] OR "Mental Disorders due to General Medical Conditions"[tw] OR "Neurosis"[tw] OR "Paraphilias"[tw] OR "Personality Disorders"[tw] OR "Pseudodementia"[tw] OR "Psychosis"[tw] OR "Schizoaffective Disorder"[tw] OR "Attention Deficit Disorder"[tw] OR "Mental Disorder"[tw] OR "Adjustment Disorder"[tw] OR "Affective Disorder"[tw] OR "Anxiety Disorder"[tw] OR "Autism"[tw] OR "autostoc"[tw] OR "Dissociative Disorder"[tw] OR "Eating Disorder"[tw] OR "Factitious Disorder"[tw] OR "Gender Identity Disorder"[tw] OR "Hoarding Disorder"[tw] OR "Impulse Control Disorder"[tw] OR "Neuroses"[tw] OR "Paraphilia"[tw] OR "Personality Disorder"[tw] OR "Psychoses"[tw] OR "Schizoaffective Disorder"[tw] OR "Attention Deficit Disorder"[tw] OR "psychiatry"[ti] OR "Child Psychiatry"[mesh] OR "Adolescent Psychiatry"[mesh] OR "child mental health"[tw] OR "adolescent mental health"[tw]))** OR (("Trust"[Mesh] OR "trust"[tw] OR trust*[tw] OR ("confidence"[tw] NOT "confidence interval"[tw]) OR "confident"[tw] OR "referral planning"[tw] OR "referral plans"[tw] OR "treatment plan"[tw] OR "treatment plans"[tw] OR "therapy plan"[tw] OR "therapy plans"[tw] OR "plan of treatment"[tw] OR "care plan"[tw] OR "care plans"[tw] OR "plan of care"[tw] OR "treatment path"[tw] OR "treatment paths"[tw] OR "therapy pathway"[tw] OR "treatment pathway"[tw] OR "treatment pathways"[tw] OR "care pathway"[tw] OR "care pathways"[tw] OR "physician satisfaction"[tw] OR "physicians satisfaction"[tw] OR "physician's satisfaction"[tw] OR "practitioner satisfaction"[tw] OR "gp satisfaction"[tw] OR "gps satisfaction"[tw] OR "doctor satisfaction"[tw] OR "doctors satisfaction"[tw] OR "doctor's satisfaction"[tw] OR "satisfied physicians"[tw] OR "satisfied gps"[tw] OR ("Personal Satisfaction"[mesh] AND "Physicians"[mesh]) OR "Practice Management"[Mesh] OR "practice management"[tw] OR "office management"[tw] OR "Practice Patterns, Physicians'"[majr] OR "Early Diagnosis"[majr] OR "early detection"[ti] OR "treatment outcome"[ti] OR "Treatment Outcome"[majr]) **AND ("Child"[mesh] OR "child"[tw] OR "children"[tw] OR "childhood"[tw] OR "child*"[tw] OR "Adolescent"[mesh] OR "adolescent"[tw] OR "adolescents"[tw] OR "adolesence"[tw] OR "adolescen*"[tw] OR "girl"[tw] OR "girls"[tw] OR "boy"[tw] OR "boys"[tw] OR "youths"[tw] OR "youth"[tw] OR "teen"[tw] OR "teens"[tw] OR "teenager"[tw] OR "teenagers"[tw]) AND ("Triage"[mesh] OR "triage"[tw] OR "Referral and Consultation"[mesh] OR "Referral"[tw] OR "referrals"[tw] OR "DAWBA"[tw] OR "Diagnostic And Well-Being Assessment"[tw] OR "Diagnostic And Wellbeing Assessment"[tw] OR "development and well being assessment"[tw] OR "development and wellbeing assessment"[tw] OR "well being assessment"[tw] OR "wellbeing assessment"[tw] OR "wellbeing assessments"[tw] OR** "Clinical decision support systems"[tw] OR "Clinical decision support system"[tw] OR "Decision Support Systems, Clinical"[mesh]**) AND ("primary health care"[majr] OR "general practitioners"[majr] OR "family practice"[majr] OR "physicians, family"[majr] OR "nurse practitioners"[majr] OR "Primary Health Care"[ti] OR "Primary Care"[ti] OR "General Practitioners"[ti] OR "Family Medicine"[ti] OR "Family Physicians"[ti] OR "General Practitioner"[ti] OR "Family Physician"[ti] OR "family doctor"[ti] OR "family doctors"[ti] OR "GP"[ti] OR "GPs"[ti] OR "General Practice"[ti] OR "family practice"[ti] OR "family practitioners"[ti] OR "family practitioner"[ti] OR "family practice"[ti] OR "nurse practitioners"[ti] OR "nurse practitioner"[ti] OR "mental health nurse practitioner"[ti] OR "mental health nurse practitioners"[ti] OR "primary mental health worker"[ti] OR "primary mental health workers"[ti] OR "first line"[ti] OR "firstline"[ti] OR "community"[ti]) AND ("community psychiatry"[mesh] OR "community mental health services"[mesh] OR "Community Psychiatry"[tw] OR "Community Mental Health"[tw] OR "Assertive Community Treatment"[tw] OR "Community Mental Health Services"[tw] OR "Community Counseling"[tw] OR "Community Psychology"[tw] OR "mental health"[mesh] OR "Mental Disorders"[mesh] OR "Mental Disorders"[tw] OR "Adjustment Disorders"[tw] OR "Affective Disorders"[tw] OR "Alexithymia"[tw] OR "Anxiety Disorders"[tw] OR "Autism Spectrum Disorders"[tw] OR "Chronic Mental Illness"[tw] OR "Dementia"[tw] OR "Dissociative Disorders"[tw] OR "Eating Disorders"[tw] OR "Elective Mutism"[tw] OR "Factitious Disorders"[tw] OR "Gender Identity Disorder"[tw] OR "Hoarding Disorder"[tw] OR "Hysteria"[tw] OR "Impulse Control Disorders"[tw] OR "Koro"[tw] OR "Mental Disorders due to General Medical Conditions"[tw] OR "Neurosis"[tw] OR "Paraphilias"[tw] OR "Personality Disorders"[tw] OR "Pseudodementia"[tw] OR "Psychosis"[tw] OR "Schizoaffective Disorder"[tw] OR "Attention Deficit Disorder"[tw] OR "Mental Disorder"[tw] OR "Adjustment Disorder"[tw] OR "Affective Disorder"[tw] OR "Anxiety Disorder"[tw] OR "Autism"[tw] OR "autostoc"[tw] OR "Dissociative Disorder"[tw] OR "Eating Disorder"[tw] OR "Factitious Disorder"[tw] OR "Gender Identity Disorder"[tw] OR "Hoarding Disorder"[tw] OR "Impulse Control Disorder"[tw] OR "Neuroses"[tw] OR "Paraphilia"[tw] OR "Personality Disorder"[tw] OR "Psychoses"[tw] OR "Schizoaffective Disorder"[tw] OR "Attention Deficit Disorder"[tw] OR "psychiatry"[ti] OR "Child Psychiatry"[mesh] OR "Adolescent Psychiatry"[mesh] OR "child mental health"[tw] OR "adolescent mental health"[tw]))** OR ("Decision Support Systems, Clinical"[majr] AND ("Algorithms"[mesh] OR "User-Computer Interface"[mesh] OR "Software Design"[mesh] OR "Computers"[mesh] OR "Attitude to Computers"[mesh] OR "Diagnosis, Computer-Assisted"[mesh] OR "Expert Systems"[mesh]) AND "Humans"[mesh] AND english[la] AND ("Depression"[majr] OR "Depressive Disorder, Major"[majr] OR "Schizophrenia"[majr] OR "Adolescent Psychiatry"[majr] OR "Psychiatry"[majr] OR "psychiatry"[tw] OR "Child Psychiatry"[majr] OR "mental health"[tw] OR "Mental Health"[majr])))

PsycINFO (<http://search.ebscohost.com/login.aspx?authtype=ip,uid&profile=lumc&defaultdb=psyh>)
**((**DE "Trust (Social Behavior)" OR (DE "Job Satisfaction" AND (DE "Physicians" OR DE "Family Physicians" OR DE "General Practitioners")) OR TI "trust" OR TI trust* OR TI "confidence" OR TI "confident" OR TI "referral planning" OR TI "referral plans" OR TI "treatment plan" OR TI "treatment plans" OR TI "therapy plan" OR TI "therapy plans" OR TI "plan of treatment" OR TI "care plan" OR TI "care plans" OR TI "plan of care" OR TI "treatment path" OR TI "treatment paths" OR TI "therapy pathway" OR TI "treatment pathway" OR TI "treatment pathways" OR TI "care pathway" OR TI "care pathways" OR TI "physician satisfaction" OR TI "physicians satisfaction" OR TI "physician's satisfaction" OR TI "practitioner satisfaction" OR TI "gp satisfaction" OR TI "gps satisfaction" OR TI "doctor satisfaction" OR TI "doctors satisfaction" OR TI "doctor's satisfaction" OR TI "satisfied physicians" OR TI "satisfied gps" OR TI "practice management" OR TI "office management" OR DE "trust" OR DE trust* OR DE "confidence" OR DE "confident" OR DE "referral planning" OR DE "referral plans" OR DE "treatment plan" OR DE "treatment plans" OR DE "therapy plan" OR DE "therapy plans" OR DE "plan of treatment" OR DE "care plan" OR DE "care plans" OR DE "plan of care" OR DE "treatment path" OR DE "treatment paths" OR DE "therapy pathway" OR DE "treatment pathway" OR DE "treatment pathways" OR DE "care pathway" OR DE "care pathways" OR DE "physician satisfaction" OR DE "physicians satisfaction" OR DE "physician's satisfaction" OR DE "practitioner satisfaction" OR DE "gp satisfaction" OR DE "gps satisfaction" OR DE "doctor satisfaction" OR DE "doctors satisfaction" OR DE "doctor's satisfaction" OR DE "satisfied physicians" OR DE "satisfied gps" OR DE "practice management" OR DE "office management" OR AB "trust" OR MA trust* OR MA "confidence" OR MA "confident" OR MA "referral planning" OR MA "referral plans" OR MA "treatment plan" OR MA "treatment plans" OR MA "therapy plan" OR MA "therapy plans" OR MA "plan of treatment" OR MA "care plan" OR MA "care plans" OR MA "plan of care" OR MA "treatment path" OR MA "treatment paths" OR MA "therapy pathway" OR MA "treatment pathway" OR MA "treatment pathways" OR MA "care pathway" OR MA "care pathways" OR MA "physician satisfaction" OR MA "physicians satisfaction" OR MA "physician's satisfaction" OR MA "practitioner satisfaction" OR MA "gp satisfaction" OR MA "gps satisfaction" OR MA "doctor satisfaction" OR MA "doctors satisfaction" OR MA "doctor's satisfaction" OR MA "satisfied physicians" OR MA "satisfied gps" OR MA "practice management" OR MA "office management" OR AB "trust" OR AB trust* OR AB "confidence" OR AB "confident" OR AB "referral planning" OR AB "referral plans" OR AB "treatment plan" OR AB "treatment plans" OR AB "therapy plan" OR AB "therapy plans" OR AB "plan of treatment" OR AB "care plan" OR AB "care plans" OR AB "plan of care" OR AB "treatment path" OR AB "treatment paths" OR AB "therapy pathway" OR AB "treatment pathway" OR AB "treatment pathways" OR AB "care pathway" OR AB "care pathways" OR AB "physician satisfaction" OR AB "physicians satisfaction" OR AB "physician's satisfaction" OR AB "practitioner satisfaction" OR AB "gp satisfaction" OR AB "gps satisfaction" OR AB "doctor satisfaction" OR AB "doctors satisfaction" OR AB "doctor's satisfaction" OR AB "satisfied physicians" OR AB "satisfied gps" OR AB "practice ABnagement" OR AB "office ABnagement" OR TI("early detection")) **AND (TI(child OR children OR childhood OR child* OR adolescent OR adolescents OR adolesence OR adolescen* OR girl OR girls OR boy OR boys OR pediatr* OR paediatr*) OR AG((Childhood (birth-12 yrs)) OR (Neonatal (birth-1 mo)) OR (Infancy (2-23 mo)) OR (Preschool Age (2-5 yrs)) OR (School Age (6-12 yrs)) OR (Adolescence (13-17 yrs)))) AND** (**TI("DAWBA" OR "Diagnostic And Well Being Assessment" OR "Diagnostic And Wellbeing Assessment" OR "development and well being assessment" OR "development and wellbeing assessment" OR "wellbeing assessment" OR "well being assessment" OR "Triage" OR "triage" OR "Referral and Consultation" OR "Referral" OR "referrals" OR "DAWBA" OR "Diagnostic And Well-Being Assessment" OR "Diagnostic And Wellbeing Assessment" OR "development and well being assessment" OR "development and wellbeing assessment" OR "well being assessment" OR "wellbeing assessment" OR "wellbeing assessments") OR DE "Professional Referral" OR DE "Client Transfer" OR TI(screen*) OR AB(screen*)) AND (TI(Primary Health Care OR Primary Care OR General Practitioners OR Family Medicine OR Family Physicians OR General Practitioner OR Family Physician OR family doctor OR family doctors OR GP OR GPs OR General Practice OR family practice OR family practitioners OR family practitioner OR family practice OR nurse practitioners OR nurse practitioner OR mental health nurse practitioner OR mental health nurse practitioners OR primary mental health worker OR primary mental health workers OR first line OR firstline) OR SU(Primary Health Care OR Primary Care OR General Practitioners OR Family Medicine OR Family Physicians OR General Practitioner OR Family Physician OR family doctor OR family doctors OR GP OR GPs OR General Practice OR family practice OR family practitioners OR family practitioner OR family practice OR nurse practitioners OR nurse practitioner OR mental health nurse practitioner OR mental health nurse practitioners OR primary mental health worker OR primary mental health workers OR first line OR firstline) OR AB(Primary Health Care OR Primary Care OR General Practitioners OR Family Medicine OR Family Physicians OR General Practitioner OR Family Physician OR family doctor OR family doctors OR GP OR GPs OR General Practice OR family practice OR family practitioners OR family practitioner OR family practice OR nurse practitioners OR nurse practitioner OR mental health nurse practitioner OR mental health nurse practitioners OR primary mental health worker OR primary mental health workers OR first line OR firstline) OR SU(Primary Health Care OR Primary Care OR General Practitioners OR Family Medicine OR Family Physicians OR General Practitioner OR Family Physician OR family doctor OR family doctors OR GP OR GPs OR General Practice OR family practice OR family practitioners OR family practitioner OR family practice OR nurse practitioners OR nurse practitioner OR mental health nurse practitioner OR mental health nurse practitioners OR primary mental health worker OR primary mental health workers OR first line OR firstline) OR (DE "General Practitioners" OR DE "Family Medicine" OR DE "Family Physicians" OR DE "Primary Health Care) AND (TI(Community Psychiatry OR Community Mental Health OR Assertive Community Treatment OR Community Mental Health Services OR Community Counseling OR Community Psychology) OR SU(Community Psychiatry OR Community Mental Health OR Assertive Community Treatment OR Community Mental Health Services OR Community Counseling OR Community Psychology) OR DE "Community Psychiatry" OR DE "Community Mental Health" OR DE "Assertive Community Treatment" OR DE "Community Mental Health Services" OR DE "Community Counseling" OR DE "Community Psychology" OR DE "Mental Disorders" OR DE "Adjustment Disorders" OR DE "Affective Disorders" OR DE "Alexithymia" OR DE "Anxiety Disorders" OR DE "Autism Spectrum Disorders" OR DE "Chronic Mental Illness" OR DE "Dementia" OR DE "Dissociative Disorders" OR DE "Eating Disorders" OR DE "Elective Mutism" OR DE "Factitious Disorders" OR DE "Gender Identity Disorder" OR DE "Hoarding Disorder" OR DE "Hysteria" OR DE "Impulse Control Disorders" OR DE "Koro" OR DE "Mental Disorders due to General Medical Conditions" OR DE "Neurosis" OR DE "Paraphilias" OR DE "Personality Disorders" OR DE "Pseudodementia" OR DE "Psychosis" OR DE "Schizoaffective Disorder" OR TI "Community Psychiatry" OR TI "Community Mental Health" OR TI "Assertive Community Treatment" OR TI "Community Mental Health Services" OR TI "Community Counseling" OR TI "Community Psychology" OR TI "Mental Disorders" OR TI "Adjustment Disorders" OR TI "Affective Disorders" OR TI "Alexithymia" OR TI "Anxiety Disorders" OR TI "Autism Spectrum Disorders" OR TI "Chronic Mental Illness" OR TI "Dementia" OR TI "Dissociative Disorders" OR TI "Eating Disorders" OR TI "Elective Mutism" OR TI "Factitious Disorders" OR TI "Gender Identity Disorder" OR TI "Hoarding Disorder" OR TI "Hysteria" OR TI "Impulse Control Disorders" OR TI "Koro" OR TI "Mental Disorders due to General Medical Conditions" OR TI "Neurosis" OR TI "Paraphilias" OR TI "Personality Disorders" OR TI "Pseudodementia" OR TI "Psychosis" OR TI "Schizoaffective Disorder" OR DE "Attention Deficit Disorder" OR DE "Attention Deficit Disorder with Hyperactivity" OR TI "Attention Deficit Disorder" OR TI "Attention Deficit Disorder with Hyperactivity"))**

OR

**((**DE "Trust (Social Behavior)" OR (DE "Job Satisfaction" AND (DE "Physicians" OR DE "Family Physicians" OR DE "General Practitioners")) OR TI "trust" OR TI trust* OR TI "confidence" OR TI "confident" OR TI "referral planning" OR TI "referral plans" OR TI "treatment plan" OR TI "treatment plans" OR TI "therapy plan" OR TI "therapy plans" OR TI "plan of treatment" OR TI "care plan" OR TI "care plans" OR TI "plan of care" OR TI "treatment path" OR TI "treatment paths" OR TI "therapy pathway" OR TI "treatment pathway" OR TI "treatment pathways" OR TI "care pathway" OR TI "care pathways" OR TI "physician satisfaction" OR TI "physicians satisfaction" OR TI "physician's satisfaction" OR TI "practitioner satisfaction" OR TI "gp satisfaction" OR TI "gps satisfaction" OR TI "doctor satisfaction" OR TI "doctors satisfaction" OR TI "doctor's satisfaction" OR TI "satisfied physicians" OR TI "satisfied gps" OR TI "practice management" OR TI "office management" OR DE "trust" OR DE trust* OR DE "confidence" OR DE "confident" OR DE "referral planning" OR DE "referral plans" OR DE "treatment plan" OR DE "treatment plans" OR DE "therapy plan" OR DE "therapy plans" OR DE "plan of treatment" OR DE "care plan" OR DE "care plans" OR DE "plan of care" OR DE "treatment path" OR DE "treatment paths" OR DE "therapy pathway" OR DE "treatment pathway" OR DE "treatment pathways" OR DE "care pathway" OR DE "care pathways" OR DE "physician satisfaction" OR DE "physicians satisfaction" OR DE "physician's satisfaction" OR DE "practitioner satisfaction" OR DE "gp satisfaction" OR DE "gps satisfaction" OR DE "doctor satisfaction" OR DE "doctors satisfaction" OR DE "doctor's satisfaction" OR DE "satisfied physicians" OR DE "satisfied gps" OR DE "practice management" OR DE "office management" OR AB "trust" OR MA trust* OR MA "confidence" OR MA "confident" OR MA "referral planning" OR MA "referral plans" OR MA "treatment plan" OR MA "treatment plans" OR MA "therapy plan" OR MA "therapy plans" OR MA "plan of treatment" OR MA "care plan" OR MA "care plans" OR MA "plan of care" OR MA "treatment path" OR MA "treatment paths" OR MA "therapy pathway" OR MA "treatment pathway" OR MA "treatment pathways" OR MA "care pathway" OR MA "care pathways" OR MA "physician satisfaction" OR MA "physicians satisfaction" OR MA "physician's satisfaction" OR MA "practitioner satisfaction" OR MA "gp satisfaction" OR MA "gps satisfaction" OR MA "doctor satisfaction" OR MA "doctors satisfaction" OR MA "doctor's satisfaction" OR MA "satisfied physicians" OR MA "satisfied gps" OR MA "practice management" OR MA "office management" OR AB "trust" OR AB trust* OR AB "confidence" OR AB "confident" OR AB "referral planning" OR AB "referral plans" OR AB "treatment plan" OR AB "treatment plans" OR AB "therapy plan" OR AB "therapy plans" OR AB "plan of treatment" OR AB "care plan" OR AB "care plans" OR AB "plan of care" OR AB "treatment path" OR AB "treatment paths" OR AB "therapy pathway" OR AB "treatment pathway" OR AB "treatment pathways" OR AB "care pathway" OR AB "care pathways" OR AB "physician satisfaction" OR AB "physicians satisfaction" OR AB "physician's satisfaction" OR AB "practitioner satisfaction" OR AB "gp satisfaction" OR AB "gps satisfaction" OR AB "doctor satisfaction" OR AB "doctors satisfaction" OR AB "doctor's satisfaction" OR AB "satisfied physicians" OR AB "satisfied gps" OR AB "practice ABnagement" OR AB "office ABnagement" OR TI("early detection")) **AND (TI(child OR children OR childhood OR child* OR adolescent OR adolescents OR adolesence OR adolescen* OR girl OR girls OR boy OR boys OR pediatr* OR paediatr*) OR AG((Childhood (birth-12 yrs)) OR (Neonatal (birth-1 mo)) OR (Infancy (2-23 mo)) OR (Preschool Age (2-5 yrs)) OR (School Age (6-12 yrs)) OR (Adolescence (13-17 yrs)))) AND (TX("DAWBA" OR "Diagnostic And Well Being Assessment" OR "Diagnostic And Wellbeing Assessment" OR "development and well being assessment" OR "development and wellbeing assessment" OR "wellbeing assessment" OR "well being assessment" OR "Triage" OR "triage" OR "Referral and Consultation" OR "Referral" OR "referrals" OR "DAWBA" OR "Diagnostic And Well-Being Assessment" OR "Diagnostic And Wellbeing Assessment" OR "development and well being assessment" OR "development and wellbeing assessment" OR "well being assessment" OR "wellbeing assessment" OR "wellbeing assessments") OR DE "Professional Referral" OR DE "Client Transfer" OR TI(screen*) OR AB(screen*)) AND (TI(Primary Health Care OR Primary Care OR General Practitioners OR Family Medicine OR Family Physicians OR General Practitioner OR Family Physician OR family doctor OR family doctors OR GP OR GPs OR General Practice OR family practice OR family practitioners OR family practitioner OR family practice OR nurse practitioners OR nurse practitioner OR mental health nurse practitioner OR mental health nurse practitioners OR primary mental health worker OR primary mental health workers OR first line OR firstline) OR SU(Primary Health Care OR Primary Care OR General Practitioners OR Family Medicine OR Family Physicians OR General Practitioner OR Family Physician OR family doctor OR family doctors OR GP OR GPs OR General Practice OR family practice OR family practitioners OR family practitioner OR family practice OR nurse practitioners OR nurse practitioner OR mental health nurse practitioner OR mental health nurse practitioners OR primary mental health worker OR primary mental health workers OR first line OR firstline) OR (DE "General Practitioners" OR DE "Family Medicine" OR DE "Family Physicians" OR DE "Primary Health Care) AND (TI(Community Psychiatry OR Community Mental Health OR Assertive Community Treatment OR Community Mental Health Services OR Community Counseling OR Community Psychology) OR SU(Community Psychiatry OR Community Mental Health OR Assertive Community Treatment OR Community Mental Health Services OR Community Counseling OR Community Psychology) OR DE "Community Psychiatry" OR DE "Community Mental Health" OR DE "Assertive Community Treatment" OR DE "Community Mental Health Services" OR DE "Community Counseling" OR DE "Community Psychology" OR DE "Mental Disorders" OR DE "Adjustment Disorders" OR DE "Affective Disorders" OR DE "Alexithymia" OR DE "Anxiety Disorders" OR DE "Autism Spectrum Disorders" OR DE "Chronic Mental Illness" OR DE "Dementia" OR DE "Dissociative Disorders" OR DE "Eating Disorders" OR DE "Elective Mutism" OR DE "Factitious Disorders" OR DE "Gender Identity Disorder" OR DE "Hoarding Disorder" OR DE "Hysteria" OR DE "Impulse Control Disorders" OR DE "Koro" OR DE "Mental Disorders due to General Medical Conditions" OR DE "Neurosis" OR DE "Paraphilias" OR DE "Personality Disorders" OR DE "Pseudodementia" OR DE "Psychosis" OR DE "Schizoaffective Disorder" OR TI "Community Psychiatry" OR TI "Community Mental Health" OR TI "Assertive Community Treatment" OR TI "Community Mental Health Services" OR TI "Community Counseling" OR TI "Community Psychology" OR TI "Mental Disorders" OR TI "Adjustment Disorders" OR TI "Affective Disorders" OR TI "Alexithymia" OR TI "Anxiety Disorders" OR TI "Autism Spectrum Disorders" OR TI "Chronic Mental Illness" OR TI "Dementia" OR TI "Dissociative Disorders" OR TI "Eating Disorders" OR TI "Elective Mutism" OR TI "Factitious Disorders" OR TI "Gender Identity Disorder" OR TI "Hoarding Disorder" OR TI "Hysteria" OR TI "Impulse Control Disorders" OR TI "Koro" OR TI "Mental Disorders due to General Medical Conditions" OR TI "Neurosis" OR TI "Paraphilias" OR TI "Personality Disorders" OR TI "Pseudodementia" OR TI "Psychosis" OR TI "Schizoaffective Disorder" OR DE "Attention Deficit Disorder" OR DE "Attention Deficit Disorder with Hyperactivity" OR TI "Attention Deficit Disorder" OR TI "Attention Deficit Disorder with Hyperactivity"))**Embase

(<http://ovidsp.ovid.com/ovidweb.cgi?T=JS&PAGE=main&MODE=ovid&D=oemezd>)

(((exp "Trust"/ OR "trust".ti,ab OR trust*.ti,ab OR "confidence".ti,ab OR "confident".ti,ab OR "referral planning".ti,ab OR "referral plans".ti,ab OR "treatment plan".ti,ab OR "treatment plans".ti,ab OR "therapy plan".ti,ab OR "therapy plans".ti,ab OR "plan of treatment".ti,ab OR "care plan".ti,ab OR "care plans".ti,ab OR "plan of care".ti,ab OR "treatment path".ti,ab OR "treatment paths".ti,ab OR "therapy pathway".ti,ab OR "treatment pathway".ti,ab OR "treatment pathways".ti,ab OR "care pathway".ti,ab OR "care pathways".ti,ab OR "physician satisfaction".ti,ab OR "physicians satisfaction".ti,ab OR "physician's satisfaction".ti,ab OR "practitioner satisfaction".ti,ab OR "gp satisfaction".ti,ab OR "gps satisfaction".ti,ab OR "doctor satisfaction".ti,ab OR "doctors satisfaction".ti,ab OR "doctor's satisfaction".ti,ab OR "satisfied physicians".ti,ab OR "satisfied gps".ti,ab OR (("Satisfaction"/ OR "Job Satisfaction"/) AND exp "Physician"/) OR "practice management".ti,ab OR "office management".ti,ab OR exp *"Early Diagnosis"/ OR "early detection".ti) **AND (exp "Child"/ OR "child".mp OR "children".mp OR "childhood".mp OR "child*".mp OR exp "Adolescent"/ OR "adolescent".mp OR "adolescents".mp OR "adolesence".mp OR adolescen*.mp OR "girl".mp OR "girls".mp OR "boy".mp OR "boys".mp OR "youths".mp OR "youth".mp OR "teen".mp OR "teens".mp OR "teenager".mp OR "teenagers".mp) AND ("triage".ti OR *"Patient Referral"/ OR "Referral".ti OR "referrals".ti OR "DAWBA".ti OR "Diagnostic And Well-Being Assessment".ti OR "Diagnostic And Wellbeing Assessment".ti OR "development and well being assessment".ti OR "development and wellbeing assessment".ti OR "well being assessment".ti OR "wellbeing assessment".ti OR "wellbeing assessments".ti OR screen*.ti) AND (exp "primary health care"/ OR "general practitioner"/ OR "general practice"/ OR "general practitioner"/ OR exp "nurse practitioners"/ OR "Primary Health Care".mp OR "Primary Care".mp OR "General Practitioners".mp OR "Family Medicine".mp OR "Family Physicians".mp OR "General Practitioner".mp OR "Family Physician".mp OR "family doctor".mp OR "family doctors".mp OR "GP".mp OR "GPs".mp OR "General Practice".mp OR "family practice".mp OR "family practitioners".mp OR "family practitioner".mp OR "family practice".mp OR "nurse practitioners".mp OR "nurse practitioner".mp OR "mental health nurse practitioner".mp OR "mental health nurse practitioners".mp OR "primary mental health worker".mp OR "primary mental health workers".mp OR "first line".mp OR "firstline".mp OR "community".mp) AND ("social psychiatry"/ OR "mental health service"/ OR "Community Psychiatry".mp OR "Community Mental Health".mp OR "Assertive Community Treatment".mp OR "Community Mental Health Services".mp OR "Community Counseling".mp OR "Community Psychology".mp OR exp "mental health"/ OR exp "Mental Disease"/ OR "Mental Disorders".ti,ab OR "Adjustment Disorders".ti,ab OR "Affective Disorders".ti,ab OR "Alexithymia".ti,ab OR "Anxiety Disorders".ti,ab OR "Autism Spectrum Disorders".ti,ab OR "Chronic Mental Illness".ti,ab OR "Dementia".ti,ab OR "Dissociative Disorders".ti,ab OR "Eating Disorders".ti,ab OR "Elective Mutism".ti,ab OR "Factitious Disorders".ti,ab OR "Gender Identity Disorder".ti,ab OR "Hoarding Disorder".ti,ab OR "Hysteria".ti,ab OR "Impulse Control Disorders".ti,ab OR "Koro".ti,ab OR "Mental Disorders due to General Medical Conditions".ti,ab OR "Neurosis".ti,ab OR "Paraphilias".ti,ab OR "Personality Disorders".ti,ab OR "Pseudodementia".ti,ab OR "Psychosis".ti,ab OR "Schizoaffective Disorder".ti,ab OR "Attention Deficit Disorder".ti,ab OR "Mental Disorder".ti,ab OR "Adjustment Disorder".ti,ab OR "Affective Disorder".ti,ab OR "Anxiety Disorder".ti,ab OR "Autism".ti,ab OR "autostoc".ti,ab OR "Dissociative Disorder".ti,ab OR "Eating Disorder".ti,ab OR "Factitious Disorder".ti,ab OR "Gender Identity Disorder".ti,ab OR "Hoarding Disorder".ti,ab OR "Impulse Control Disorder".ti,ab OR "Neuroses".ti,ab OR "Paraphilia".ti,ab OR "Personality Disorder".ti,ab OR "Psychoses".ti,ab OR "Schizoaffective Disorder".ti,ab OR "Attention Deficit Disorder".ti,ab))** OR ((exp "Trust"/ OR "trust".ti,ab OR trust*.ti,ab OR "confidence".ti,ab OR "confident".ti,ab OR "referral planning".ti,ab OR "referral plans".ti,ab OR "treatment plan".ti,ab OR "treatment plans".ti,ab OR "therapy plan".ti,ab OR "therapy plans".ti,ab OR "plan of treatment".ti,ab OR "care plan".ti,ab OR "care plans".ti,ab OR "plan of care".ti,ab OR "treatment path".ti,ab OR "treatment paths".ti,ab OR "therapy pathway".ti,ab OR "treatment pathway".ti,ab OR "treatment pathways".ti,ab OR "care pathway".ti,ab OR "care pathways".ti,ab OR "physician satisfaction".ti,ab OR "physicians satisfaction".ti,ab OR "physician's satisfaction".ti,ab OR "practitioner satisfaction".ti,ab OR "gp satisfaction".ti,ab OR "gps satisfaction".ti,ab OR "doctor satisfaction".ti,ab OR "doctors satisfaction".ti,ab OR "doctor's satisfaction".ti,ab OR "satisfied physicians".ti,ab OR "satisfied gps".ti,ab OR (("Satisfaction"/ OR "Job Satisfaction"/) AND exp "Physician"/) OR "practice management".ti,ab OR "office management".ti,ab OR exp *"Early Diagnosis"/ OR "early detection".ti) **AND (exp "Child"/ OR "child".mp OR "children".mp OR "childhood".mp OR "child*".mp OR exp "Adolescent"/ OR "adolescent".mp OR "adolescents".mp OR "adolesence".mp OR adolescen*.mp OR "girl".mp OR "girls".mp OR "boy".mp OR "boys".mp OR "youths".mp OR "youth".mp OR "teen".mp OR "teens".mp OR "teenager".mp OR "teenagers".mp) AND ("triage".mp OR "Patient Referral"/ OR "Referral".mp OR "referrals".mp OR "DAWBA".mp OR "Diagnostic And Well-Being Assessment".mp OR "Diagnostic And Wellbeing Assessment".mp OR "development and well being assessment".mp OR "development and wellbeing assessment".mp OR "well being assessment".mp OR "wellbeing assessment".mp OR "wellbeing assessments".mp OR screen*.ti) AND (exp *"primary health care"/ OR *"general practitioner"/ OR *"general practice"/ OR *"general practitioner"/ OR exp *"nurse practitioners"/ OR "Primary Health Care".ti OR "Primary Care".ti OR "General Practitioners".ti OR "Family Medicine".ti OR "Family Physicians".ti OR "General Practitioner".ti OR "Family Physician".ti OR "family doctor".ti OR "family doctors".ti OR "GP".ti OR "GPs".ti OR "General Practice".ti OR "family practice".ti OR "family practitioners".ti OR "family practitioner".ti OR "family practice".ti OR "nurse practitioners".ti OR "nurse practitioner".ti OR "mental health nurse practitioner".ti OR "mental health nurse practitioners".ti OR "primary mental health worker".ti OR "primary mental health workers".ti OR "first line".ti OR "firstline".ti OR "community".ti) AND ("social psychiatry"/ OR "mental health service"/ OR "Community Psychiatry".mp OR "Community Mental Health".mp OR "Assertive Community Treatment".mp OR "Community Mental Health Services".mp OR "Community Counseling".mp OR "Community Psychology".mp OR exp "mental health"/ OR exp "Mental Disease"/ OR "Mental Disorders".ti,ab OR "Adjustment Disorders".ti,ab OR "Affective Disorders".ti,ab OR "Alexithymia".ti,ab OR "Anxiety Disorders".ti,ab OR "Autism Spectrum Disorders".ti,ab OR "Chronic Mental Illness".ti,ab OR "Dementia".ti,ab OR "Dissociative Disorders".ti,ab OR "Eating Disorders".ti,ab OR "Elective Mutism".ti,ab OR "Factitious Disorders".ti,ab OR "Gender Identity Disorder".ti,ab OR "Hoarding Disorder".ti,ab OR "Hysteria".ti,ab OR "Impulse Control Disorders".ti,ab OR "Koro".ti,ab OR "Mental Disorders due to General Medical Conditions".ti,ab OR "Neurosis".ti,ab OR "Paraphilias".ti,ab OR "Personality Disorders".ti,ab OR "Pseudodementia".ti,ab OR "Psychosis".ti,ab OR "Schizoaffective Disorder".ti,ab OR "Attention Deficit Disorder".ti,ab OR "Mental Disorder".ti,ab OR "Adjustment Disorder".ti,ab OR "Affective Disorder".ti,ab OR "Anxiety Disorder".ti,ab OR "Autism".ti,ab OR "autostoc".ti,ab OR "Dissociative Disorder".ti,ab OR "Eating Disorder".ti,ab OR "Factitious Disorder".ti,ab OR "Gender Identity Disorder".ti,ab OR "Hoarding Disorder".ti,ab OR "Impulse Control Disorder".ti,ab OR "Neuroses".ti,ab OR "Paraphilia".ti,ab OR "Personality Disorder".ti,ab OR "Psychoses".ti,ab OR "Schizoaffective Disorder".ti,ab OR "Attention Deficit Disorder".ti,ab)))**Web Of Science **(**<http://isiknowledge.com/wos>)

((ts=("Trust" OR "trust" OR trust* OR "confidence" OR "confident" OR "referral planning" OR "referral plans" OR "treatment plan" OR "treatment plans" OR "therapy plan" OR "therapy plans" OR "plan of treatment" OR "care plan" OR "care plans" OR "plan of care" OR "treatment path" OR "treatment paths" OR "therapy pathway" OR "treatment pathway" OR "treatment pathways" OR "care pathway" OR "care pathways" OR "physician satisfaction" OR "physicians satisfaction" OR "physician's satisfaction" OR "practitioner satisfaction" OR "gp satisfaction" OR "gps satisfaction" OR "doctor satisfaction" OR "doctors satisfaction" OR "doctor's satisfaction" OR "satisfied physicians" OR "satisfied gps" OR (("Satisfaction" OR "Job Satisfaction") AND "Physician") OR "practice management" OR "office management" OR "Early Diagnosis" OR "early detection") **AND ts=("Child" OR "child" OR "children" OR "childhood" OR "child*" OR "Adolescent" OR "adolescent" OR "adolescents" OR "adolesence" OR adolescen* OR "girl" OR "girls" OR "boy" OR "boys" OR "youths" OR "youth" OR "teen" OR "teens" OR "teenager" OR "teenagers") AND ti=("triage" OR *"Patient Referral" OR "Referral" OR "referrals" OR "DAWBA" OR "Diagnostic And Well-Being Assessment" OR "Diagnostic And Wellbeing Assessment" OR "development and well being assessment" OR "development and wellbeing assessment" OR "well being assessment" OR "wellbeing assessment" OR "wellbeing assessments" OR screen*) AND ts=("primary health care" OR "general practitioner" OR "general practice" OR "general practitioner" OR "nurse practitioners" OR "Primary Health Care" OR "Primary Care" OR "General Practitioners" OR "Family Medicine" OR "Family Physicians" OR "General Practitioner" OR "Family Physician" OR "family doctor" OR "family doctors" OR "GP" OR "GPs" OR "General Practice" OR "family practice" OR "family practitioners" OR "family practitioner" OR "family practice" OR "nurse practitioners" OR "nurse practitioner" OR "mental health nurse practitioner" OR "mental health nurse practitioners" OR "primary mental health worker" OR "primary mental health workers" OR "first line" OR "firstline" OR "community") AND ts=("social psychiatry" OR "mental health service" OR "Community Psychiatry" OR "Community Mental Health" OR "Assertive Community Treatment" OR "Community Mental Health Services" OR "Community Counseling" OR "Community Psychology" OR "mental health" OR "Mental Disease" OR "Mental Disorders" OR "Adjustment Disorders" OR "Affective Disorders" OR "Alexithymia" OR "Anxiety Disorders" OR "Autism Spectrum Disorders" OR "Chronic Mental Illness" OR "Dementia" OR "Dissociative Disorders" OR "Eating Disorders" OR "Elective Mutism" OR "Factitious Disorders" OR "Gender Identity Disorder" OR "Hoarding Disorder" OR "Hysteria" OR "Impulse Control Disorders" OR "Koro" OR "Mental Disorders due to General Medical Conditions" OR "Neurosis" OR "Paraphilias" OR "Personality Disorders" OR "Pseudodementia" OR "Psychosis" OR "Schizoaffective Disorder" OR "Attention Deficit Disorder" OR "Mental Disorder" OR "Adjustment Disorder" OR "Affective Disorder" OR "Anxiety Disorder" OR "Autism" OR "autostoc" OR "Dissociative Disorder" OR "Eating Disorder" OR "Factitious Disorder" OR "Gender Identity Disorder" OR "Hoarding Disorder" OR "Impulse Control Disorder" OR "Neuroses" OR "Paraphilia" OR "Personality Disorder" OR "Psychoses" OR "Schizoaffective Disorder" OR "Attention Deficit Disorder"))** OR (ts=("Trust" OR "trust" OR trust* OR "confidence" OR "confident" OR "referral planning" OR "referral plans" OR "treatment plan" OR "treatment plans" OR "therapy plan" OR "therapy plans" OR "plan of treatment" OR "care plan" OR "care plans" OR "plan of care" OR "treatment path" OR "treatment paths" OR "therapy pathway" OR "treatment pathway" OR "treatment pathways" OR "care pathway" OR "care pathways" OR "physician satisfaction" OR "physicians satisfaction" OR "physician's satisfaction" OR "practitioner satisfaction" OR "gp satisfaction" OR "gps satisfaction" OR "doctor satisfaction" OR "doctors satisfaction" OR "doctor's satisfaction" OR "satisfied physicians" OR "satisfied gps" OR (("Satisfaction" OR "Job Satisfaction") AND "Physician") OR "practice management" OR "office management" OR "Early Diagnosis" OR "early detection") **AND ts=("Child" OR "child" OR "children" OR "childhood" OR "child*" OR "Adolescent" OR "adolescent" OR "adolescents" OR "adolesence" OR adolescen* OR "girl" OR "girls" OR "boy" OR "boys" OR "youths" OR "youth" OR "teen" OR "teens" OR "teenager" OR "teenagers") AND (ts=("triage" OR "Patient Referral" OR "Referral" OR "referrals" OR "DAWBA" OR "Diagnostic And Well-Being Assessment" OR "Diagnostic And Wellbeing Assessment" OR "development and well being assessment" OR "development and wellbeing assessment" OR "well being assessment" OR "wellbeing assessment" OR "wellbeing assessments") OR ti=screen*) AND ti=("primary health care" OR *"general practitioner" OR *"general practice" OR *"general practitioner" OR "nurse practitioners" OR "Primary Health Care" OR "Primary Care" OR "General Practitioners" OR "Family Medicine" OR "Family Physicians" OR "General Practitioner" OR "Family Physician" OR "family doctor" OR "family doctors" OR "GP" OR "GPs" OR "General Practice" OR "family practice" OR "family practitioners" OR "family practitioner" OR "family practice" OR "nurse practitioners" OR "nurse practitioner" OR "mental health nurse practitioner" OR "mental health nurse practitioners" OR "primary mental health worker" OR "primary mental health workers" OR "first line" OR "firstline" OR "community") AND ts=("social psychiatry" OR "mental health service" OR "Community Psychiatry" OR "Community Mental Health" OR "Assertive Community Treatment" OR "Community Mental Health Services" OR "Community Counseling" OR "Community Psychology" OR "mental health" OR "Mental Disease" OR "Mental Disorders" OR "Adjustment Disorders" OR "Affective Disorders" OR "Alexithymia" OR "Anxiety Disorders" OR "Autism Spectrum Disorders" OR "Chronic Mental Illness" OR "Dementia" OR "Dissociative Disorders" OR "Eating Disorders" OR "Elective Mutism" OR "Factitious Disorders" OR "Gender Identity Disorder" OR "Hoarding Disorder" OR "Hysteria" OR "Impulse Control Disorders" OR "Koro" OR "Mental Disorders due to General Medical Conditions" OR "Neurosis" OR "Paraphilias" OR "Personality Disorders" OR "Pseudodementia" OR "Psychosis" OR "Schizoaffective Disorder" OR "Attention Deficit Disorder" OR "Mental Disorder" OR "Adjustment Disorder" OR "Affective Disorder" OR "Anxiety Disorder" OR "Autism" OR "autostoc" OR "Dissociative Disorder" OR "Eating Disorder" OR "Factitious Disorder" OR "Gender Identity Disorder" OR "Hoarding Disorder" OR "Impulse Control Disorder" OR "Neuroses" OR "Paraphilia" OR "Personality Disorder" OR "Psychoses" OR "Schizoaffective Disorder" OR "Attention Deficit Disorder")))**

**NOT ti=(veterinary OR rabbit OR rabbits OR animal OR animals OR mouse OR mice OR rodent OR rodents OR rat OR rats OR pig OR pigs OR porcine OR horse* OR equine OR cow OR cows OR bovine OR goat OR goats OR sheep OR ovine OR canine OR dog OR dogs OR feline OR cat OR cats))**

**COCHRANE (**<https://www.cochranelibrary.com/>)
(("Trust" OR "trust" OR trust* OR "confidence" OR "confident" OR "referral planning" OR "referral plans" OR "treatment plan" OR "treatment plans" OR "therapy plan" OR "therapy plans" OR "plan of treatment" OR "care plan" OR "care plans" OR "plan of care" OR "treatment path" OR "treatment paths" OR "therapy pathway" OR "treatment pathway" OR "treatment pathways" OR "care pathway" OR "care pathways" OR "physician satisfaction" OR "physicians satisfaction" OR "physician's satisfaction" OR "practitioner satisfaction" OR "gp satisfaction" OR "gps satisfaction" OR "doctor satisfaction" OR "doctors satisfaction" OR "doctor's satisfaction" OR "satisfied physicians" OR "satisfied gps" OR (("Satisfaction" OR "Job Satisfaction") AND "Physician") OR "practice management" OR "office management" OR "Early Diagnosis" OR "early detection") **AND ("Child" OR "child" OR "children" OR "childhood" OR "child*" OR "Adolescent" OR "adolescent" OR "adolescents" OR "adolesence" OR adolescen* OR "girl" OR "girls" OR "boy" OR "boys" OR "youths" OR "youth" OR "teen" OR "teens" OR "teenager" OR "teenagers") AND ("triage" OR "Patient Referral" OR "Referral" OR "referrals" OR "DAWBA" OR "Diagnostic And Well-Being Assessment" OR "Diagnostic And Wellbeing Assessment" OR "development and well being assessment" OR "development and wellbeing assessment" OR "well being assessment" OR "wellbeing assessment" OR "wellbeing assessments" OR screen*) AND ("primary health care" OR "general practitioner" OR "general practice" OR "general practitioner" OR "nurse practitioners" OR "Primary Health Care" OR "Primary Care" OR "General Practitioners" OR "Family Medicine" OR "Family Physicians" OR "General Practitioner" OR "Family Physician" OR "family doctor" OR "family doctors" OR "GP" OR "GPs" OR "General Practice" OR "family practice" OR "family practitioners" OR "family practitioner" OR "family practice" OR "nurse practitioners" OR "nurse practitioner" OR "mental health nurse practitioner" OR "mental health nurse practitioners" OR "primary mental health worker" OR "primary mental health workers" OR "first line" OR "firstline" OR "community") AND ("social psychiatry" OR "mental health service" OR "Community Psychiatry" OR "Community Mental Health" OR "Assertive Community Treatment" OR "Community Mental Health Services" OR "Community Counseling" OR "Community Psychology" OR "mental health" OR "Mental Disease" OR "Mental Disorders" OR "Adjustment Disorders" OR "Affective Disorders" OR "Alexithymia" OR "Anxiety Disorders" OR "Autism Spectrum Disorders" OR "Chronic Mental Illness" OR "Dementia" OR "Dissociative Disorders" OR "Eating Disorders" OR "Elective Mutism" OR "Factitious Disorders" OR "Gender Identity Disorder" OR "Hoarding Disorder" OR "Hysteria" OR "Impulse Control Disorders" OR "Koro" OR "Mental Disorders due to General Medical Conditions" OR "Neurosis" OR "Paraphilias" OR "Personality Disorders" OR "Pseudodementia" OR "Psychosis" OR "Schizoaffective Disorder" OR "Attention Deficit Disorder" OR "Mental Disorder" OR "Adjustment Disorder" OR "Affective Disorder" OR "Anxiety Disorder" OR "Autism" OR "autostoc" OR "Dissociative Disorder" OR "Eating Disorder" OR "Factitious Disorder" OR "Gender Identity Disorder" OR "Hoarding Disorder" OR "Impulse Control Disorder" OR "Neuroses" OR "Paraphilia" OR "Personality Disorder" OR "Psychoses" OR "Schizoaffective Disorder" OR "Attention Deficit Disorder"))**
