# Supplementary material for: Clinical decision support methods for children and youths with mental health disorders in primary care
Source: Fam Pract. 2022 Jun 3;39(6):1135–43. doi: 10.1093/fampra/cmac051 (PMC9680662; doi:10.1093/fampra/cmac051)
Supplement: cmac051_suppl_Supplementary_Table_1 [file cmac051_suppl_supplementary_table_1.docx]

| **Table 1: Functionalities and content of clinical decision-support methods described in the included studies (N=25)** | | | | | | | |  |
| --- | --- | --- | --- | --- | --- | --- | --- | --- |
| **Study (authors, year)** | **Name** | **Target** | **Target children/youth** | **Targeted professionals** | **Goal** | **Content** | **Organization** | **CDM-phase(s) which CDSM supports^*^** |
| **Buckingham (2015)** | MyGRaCE | Mental health (in general), associated risks of suicide, self-harm, harm to others, self-neglect, and vulnerability | Children/young persons who comply to criteria of targeted disorder(s) | Practitioners, type n.e.m.i.t. | To help service users assess and manage their own mental health collaboratively with practitioners | Decision support system which integrates service user and practitioner expertise about a variety of mental health areas. Data is collected as the service user answers questions based on a tree structure. Usage by practitioners requires a training workshop | Computer based decision-support methods | Diagnosis, assessment of severity |
| **Bauer (2015)** | Child Health Improvement through Computer Automation (CHICA) – ASD-module | Autism Spectrum Disorder (ASD) | Children of 18 years or younger with risk of ASD | PCPs | To improve management of ASD | Computer decision support system promoting adherence to clinical guidelines. It is designed to automate various aspects of preventive care and chronic disease management in the busy workflow of pediatric practice. CHICA produces a pre-screener form containing 20 health risk questions selected based on information in the child’s EHR, which is completed by the parent or child (>12 years) prior to seeing the PCP. The form is scanned, after which a physician worksheet is generated which contains 6 prompts to guide PCP decision-making. Added ASD-module consists of validated screening questions specifically for ASD and it provides referral support | Computer decision support system and electronic health record (EHR) | Diagnosis, assessment of severity and management^**^ |
| **Downs (2019)** | See Bauer (2015) – ASD module | Autism spectrum disorder (ASD) | Children aged 18 to 24 months | Pediatricians | To screen for autism spectrum disorders | The system communicates with the underlying electronic health record so that when a patient registers for care, CHICA analyzes the child’s record (demographic characteristics, morphometric characteristics, diagnoses and medications) and selects the highest priority 20 yes or no questions, covering a wide range of primary care issues to ask the family. These are displayed on a sheet of scannable paper or an electronic tablet that is given to the family to complete in the waiting room | Computer-based decision support intervention | See Bauer (2015) |
| **Carrol (2013)** | See Bauer (2015) – ADHD-module | Attention Deficit/Hyperactivity Disorder (ADHD) | Children between 5-12 years old at risk for ADHD | PCPs | To assist physicians in the diagnosis and treatment of ADHD | See Bauer (2015). Added ADHD-module consists of screening questions following an algorithm based on the American Academy of Pediatrics. The module provides suggestions for ADHD-diagnosis, medication adjustments, mental health referrals and visits | See Bauer (2015) | See Bauer (2015) |
| **Fortney (2013)** | Net Decision Support System (NetDSS) | Primarily depression severity, suicide risk, secondarily cognitive impairment, generalized anxiety, panic, posttraumatic stress disorders and mania | Young people with depression symptoms and risk for suicide (approximately <5% of total study population was between 18-24 years old) | Depression care managers, who facilitate communication with e.g. PCPs and their patients | To provide evidence-based depression care management | NetDSS guides the care manager through a real-time self-documenting patient encounter using evidence based-scripts, self-scoring instruments, and clinical algorithms to identify new trials, treatment phases, and outcome milestones such as nonadherence, treatment response, remission, and relapse. It has the following functional capabilities: patient registry, patient encounter scheduler, trial management, clinical decision support, progress note generator, and workload and outcomes report generator | Web-based clinical decision support system | Diagnosis, management |
| **Goodman (2000)** | Development And Well-Being Assessment (DAWBA) | Among others ADHD, emotional, conduct, oppositional, posttraumatic stress, obsessive-compulsive, oppositional-defiant and conduct disorders. Separation  anxiety, specific and social phobia, generalized anxiety and depression | Children and adolescents of 5-16 years old, susceptible for a variety of diagnoses | Child’s clinician(s) | To generate ICD-10 and DSM-IV psychiatric diagnoses | DAWBA contains a package of questionnaires, interviews and rating techniques. Parents, teacher and children (if 11-16 years old) complete a structured interview after which parents can describe the problems in an open-ended questions-section. The different sorts of information are brought together by a computer program which produces summary sheets. Experienced clinical raters can use these sheets to accept or overturn the likely diagnoses generated by the computer. DAWBA requires little training, by reviewing online materials | Integrated computer based package of measures consisting of quantitative and qualitative information | Diagnosis, assessment of severity, management (n.e.m.i.t.) |
| **Ford (2013)** | See Goodman (2000) | Emotional, behavioural, autism spectrum, attention deficit hyper-activity, eating, feeding and tic disorders. Attachment issues | Children of 5-10 years, susceptible for a variety of diagnoses | See Goodman (2000) | See Goodman (2000) | See Goodman (2000) | See Goodman (2000) | See Goodman (2000) |
|  |  |  |  |  |  |  |  |  |
| **McEwen (2016)** | DAWBA-ASD-section | Autism Spectrum Disorder (ASD) | Children and adolescents in community mental health settings, at risk for ASD (all study partici-pants were aged 8-16 years) | See Goodman (2000) | See Goodman (2000). In addition: to diagnose ASD | See Goodman (2000). In addition: the ASD module gathers information required to diagnose ASD. It can be completed by parents online or by interview and takes approximately 20 minutes to complete | See Goodman (2000) | See Goodman (2000) |
| **Moya (2005)** | DAWBA-ED-section | Eating disorders (ED), such as anorexia, bulimia nervosa and partial syndromes | Children and adolescents at risk for ED, especially girls (7-17 years old) | See Goodman (2000) | See Goodman (2000). In addition: to generate DSM-IV and ICD-10 based diagnoses regarding eating disorders | See Goodman (2000). In addition: The ED-section begins with structured questions about eating-related symptomatology and its impact on the child’s life. If definite symptoms are identified by the structured questions, clinicians use semi-structured open-ended questions to get respondents to describe the problems in their own words | See Goodman (2000) | See Goodman (2000) |
| **Robinson (2018)** | COMPASS (NAVIGATE) | First episode of psychosis | Patients aged 15 to 35 years (70.3% of total study population was 24 years or younger) | Prescribers | To facilitate patient-prescriber communication | Participants enter information about symptoms, side effects, treatment preferences, medication adherence and attitudes, and substance use into the system before consultation. Vital signs data and laboratory test results are entered. Data is summarized by the COMPASS program for review by the prescriber at the beginning of each medication visit. Integrating participant treatment priorities and the prescriber’s assessments, COMPASS provided suggested guideline treatment, such as monthly assessments in the first two years of treatment | Computerized clinical decision making tool | Diagnosis, management |
| **Reid (2013)** | Mobiletype clinical assistant tool | Mental health symptoms (in general) | Young people with mild or more severe emotional/mental health issues (14-24 years old) | General practitioners | To assist in general practitioners’ assessment and management | The *mobiletype* program is a mobile phone mental health assessment and management application which monitors mood, stress and everyday activities over eight areas of functioning, then transmits this information to general practitioners via a secure website in summary format for medical review | Mobile phone application | Diagnosis (n.e.m.i.t.), assessment of severity |
| **Fletcher (2019 and 2021)** | Link-me (randomized controlled trial) | Depression and anxiety symptoms | Youth aged 18-25, adults 26-75 years (19.5% of study partici-pants were aged 24 years or younger) | General practitioners | To test whether a patient-completed Decision Support Tool, which predicts future severity of depression and anxiety symptoms and triages individuals into care accordingly, is clinically effective and cost-effective relative to usual care | The first component of Link-me is a brief patient-completed decision support tool that draws on an individual’s responses to 23 items to predict their anxiety and depressive symptom trajectory over the next 3 months and stratify them into one of three prognostic groups. The second component is a recommendation for treatment pathways, which depend on the predicted symptom severity | A model of Stepped Mental Health Care, a patient-completed decision support tool | Diagnosis, assessment of severity and management |
| **Parker (2020)** | Youth StepCare | Depression and anxiety | Youth patients aged 14 to 17 years | General practitioners | To identify youth with unidentified symptoms of mental illness | The service consists of three components: screening, treatment recommendations and patient monitoring. In the first component, practice staff offer a mobile tablet to patients and parent upon arrival for a GP appointment, patient completes the questionnaire on a mobile tablet in the waiting room (approximately 3 minutes). In the second component, symptom scores and clinical recommendations are sent to the GP’s medical inbox, the GP discusses the results and forms a treatment plan with their patient. In the third component, fortnightly questionnaires are sent to patients who screened at mild, moderate or severe at baseline, results of monitoring questionnaires are sent to GP for review and follow-up | Web-based universal screening service on a mobile tablet | Diagnosis, assessment of severity and management |
| **Kaye (2017)** | Child and Adolescent Psychiatrists Primary Care program (CAP PC) | Mild-moderate mental health problems | Children and adolescents aged 5 to 21 years | PCPs | To increase ability to assess and manage. To promote collaboration and integration of health and mental health services | Formal education of PCPs, consultation support by phone, assistance with linkage/referral and face-to-face evaluations in selected situations. CAP PC is unique in its collaboration among 5 university-based child psychiatry divisions. All CAP PC programs are provided free of charge to PCPs and include CME credit | Site teams with 2-3 senior child and adolescent psychiatrists, and 1 liaison coordinator | Diagnosis, assessment of severity and management |
| **Gadomski (2014)** | Training and Education for the Advancement of Children’s Health (Project TEACH) | ADHD, depressive, anxiety and bipolar disorders; psychosis, sleep problems | Children with behavioural or emotional issues (age group n.e.m.i.t.) | PCPs | To provide training, consultation and referral support to build child and adolescent mental health expertise among primary care providers | Project TEACH refers to two programs, Child and Adolescent Psychiatry Education and Support Program for Primary Care Physician (CAPES) and CAP PC, that have similar aims but differ in scale, structure and service areas. Both offer free training, telephone consultations to PCPs, advice on referrals, and the ability to provide face-to-face evaluations if necessary. In both programs, calls from PCPs are handled by a central number and coverage is provided on a rotating basis | Combined program which integrates mental health services with primary care | Diagnosis, assessment of severity (n.e.m.i.t.) and management |
| **Kerker (2015)** | See Gadomski (2014). Study focusses on CAP PC | ADHD, psychosis, depression, anxiety, bipolar disorders | Children aged 0 to 21 years | PCPs | To correctly identify paediatric behavioural problems, effectively manage psychopharmacology and create and implement treatment plans by linking to existing resources | See Gadomski (2014). In addition: CAP PC consists of 15 hours in-person training, web-based learning tools, followed by a six-month distance learning program | See Gadomski (2014) | See Gadomski (2014) |
| **Yellowlees (2008)** | e-Mental Health (eMH) | Mood, anxiety, psychotic, pervasive developmental, conduct and impulse control, attention-deficit/hyperactivity, adjustment, substance abuse and cognitive disorders. Mental retardation and childhood, emotional disturbances | Children and adolescents younger than 18 years | PCPs | To provide multidisciplinary consultations by videoconference, telephone, and e-mail as well as provider education | In the eMH-program a consulting specialist meets with the patient who have been referred to UC Davis Medical Center via videoconferencing. At the end of the session, they would be joined by their PCP to discuss options, such as psychoeducation on medication, illness and diagnostic issues, exercise and lifestyle issues; a referral for therapy or testing if needed | Consultation-liaison model between PCPs, psychiatrists and a clinical psychologist | Diagnosis, assessment of severity and management |
| **Epstein (2007)** | Collaborative consultation treatment service | Attention Deficit/Hyperactivity Disorder (ADHD)-related symptoms | Stimulant-naïve children in first through fifth grades with an ADHD-related problem (mean age was 7.8 years) | Community-based physicians, pediatricians | To promote the use of titration trials and periodic monitoring during medication maintenance | Physicians are taught to prescribe 4 different weekly dosages of stimulant medication during a titration trial. These packaged medications were made available through local pharmacies. Weekly behavioural and side effect rating scales from parents and teachers are sent to a psychiatrist with ADHD-expertise who determined a best starting dosage of medication. A report describing the titration results, behavioural deteriorations or appearance of side effects (if this is the case) is sent back to physicians | Collaborative consultative service between community pediatricians and psychiatrists | Assessment of severity, management |
| **Williams (2006)** | Consultation-liaison | Mental health problems (in general) | Children | General practitioners | To assist general practitioners in making diagnoses and management plans | Psychiatrists participate in a roster and are on call to answer phone calls from general practitioners and to provide them with advice within 24 hours. In addition, if general practitioner, patient and psychiatrist agreed on this pathway, psychiatrists see the patient for face-to-face assessment within 14 days, with feedback to the general practitioner in a short time frame, preferably by an immediate telephone call, with written feedback within 14 days | Consultation-liaison service to general practitioners, provided by a cohort of private and public psychiatrists | Diagnosis, assessment of severity and management |
| **Jacob (2012)** | Telepsychiatry | Among others, major depressive disorder, generalized anxiety, seasonal affective and oppositional defiant disorders, ADHD; anxiety not otherwise specified | Children susceptible for a variety of diagnoses (aged 4-18 years) | PCPs | To diagnose and treat a wide range of psychiatric disorders, and to increase access to psychologists, psychiatrists and other therapists | A psychiatrist sees a child via videoconferencing for a limited number of sessions and then provides a treatment plan to that child’s PCP and family | A telepsychiatry consultation practice between psychiatrist and PCP | Diagnosis, assessment of severity and management |
| **Walter (2019)** | Behavioral Health Integration Program | Common psychiatric disorders, such as anxiety, depression and ADHD | Children (median age was 11 years) | PCPs | To provide in-depth behavioural health education, on-demand psychiatric consultation, operational and clinical support for integrated practice transformation and on-site clinical behavioural health service | The behavioural health integration program consists of the following components: an education component which comprised 10 sessions (16 hours), a consultation component which provides real-time telephone consultation by child and adolescent psychiatrists, a transformation component focussing on i.a. clinical and business workflows and electronic health record documentation, and an on-site clinical behavioural health service focussing on screening, guided self-management and psychopharmacology | Multicomponent, transdiagnostic integrated behavioural health model | Diagnosis, assessment of severity and management |
| **Malas (2019)** | Michigan Child Collaborative Care (MC3) Program | Among others, ADHD and mood disorders | Children and youths under the age of 24 | PCPs | To offer embedded behavioural health consultants within primary care practices, telephonic consultation, video consultation and embedded care | This program offers several levels of consultation and collaboration: behavioural health consultants who can provide referrals to local resources, brief in-person consultation, evaluation and non-pharmacologic intervention; formal telephone consultation with child and adolescent psychiatrists within the same business day of consult placement; videoconferencing with patients and families for more comprehensive telepsychiatric consultation; group case consultation for a group of PCPs in several areas of the state who wish to learn and discuss a series of cases together; and opportunities for embedded psychiatric care or in-person consultation | Telepsychiatry service | Diagnosis (n.e.m.i.t.), assessment of severity and management |
| **Thompson (2019)** | Collaborative care for depression intervention | Depression | Adolescents (12-25 years), adults (26->75 years) | PCPs | To support PCPs with screening, referral and treatment uptake of depressive symptoms | Patients are screened with the Patient Health Questionnaire-2 and the Patient Health Questionnaire-9 on the basis of an eligibility algorithm. Electronic health record data are used for sample characteristics, screening rates, referrals and treatment pathways | See column ‘Name’ | Diagnosis, assessment of severity and management |
| **Campbell (2021)** | Process changes of universal screening for autism | Autism Spectrum Disorder (ASD) | Patients aged 16 to 30 months | Resident and attending pediatricians | To identify children at risk for ASD who may be referred for further evaluation | The process change consisted of three phases. Phase 1 was changing the screening instrument (from M-CHAT-R to the POSI) and adding decision support. Phase 2 was adding automatic reminders. Phase 3 was adding a referral option for autism evaluations in primary care | A combination of a shorter and more sensitive screening instrument, staff training, clinical decision support, electronic health record automatic reminders, and primary care integration of autism evaluations | Diagnosis, management |
| Legend PCP = Primary Care Physician CDSM = Clinical Decision Support Method N.e.m.i.t. = Not Explicitly Mentioned in Text ^*^ According to Bajaj (2011). ^**^ Including referral | | | | | | | |  |
