# Supplementary material for: Clinical decision support methods for children and youths with mental health disorders in primary care
Source: Fam Pract. 2022 Jun 3;39(6):1135–43. doi: 10.1093/fampra/cmac051 (PMC9680662; doi:10.1093/fampra/cmac051)
Supplement: cmac051_suppl_Supplementary_Table_2 [file cmac051_suppl_supplementary_table_2.docx]

| **Table 2: Studies supporting clinical decision-making methods for mental health disorders in children and youths in primary care (N=25)** | | | | | | | | | | | | | | |
| --- | --- | --- | --- | --- | --- | --- | --- | --- | --- | --- | --- | --- | --- | --- |
| **Study (authors, year)** | **Objectives** | **Methods** | **Outcomes (provider)** | **Outcomes (patient)** | **Measurement moments** | **Intervention** | **Control** | **Targeted group** | **(N) Study partici-pants** | **% Gender female (provi-der)** | **% Gender female (pa-tient)** | **Mean age (pa-tient)** | **Results**  **(provider)** | **Results (patient)** |
| **Bucking-ham (2015)** | To describe the development of a CDSS that integrates service user and practitioner expertise | Interviews, focus groups, agile software develop-ment and implemen-tation of MyGRaCE | Satisfaction regarding MyGRaCE | Patient ability to assess their (un)safety, life changes, view of others, symptoms of unease, joint clinical decision-making and self-care | N.e.m.i.t. | MyGRaCE | N.a. | Service users with risk of suicide, self-harm, harm to others, self-neglect and vulne-rability | 115 service users | N.e.m. i.t. | N.e.m. i.t. | N.e.m. i.t. | Despite differences in assessing risks and safety, there was improved shared understanding of mental health risk between patients and practitioners | A challenge was how to pro-vide flexible access without over-whel-ming and con-fusing users |
| **Bauer (2015)** | To add an Autism Spectrum Disorder (ASD)-modu-le to an exis-ting CDSS | Cross-sectional survey | PCP knowledge, beliefs and self-reported practice related to ASD | N.a. | 0, 12, and 24 months after implementation. | ASD-module for CHICA | CHICA system without ASD-module | PCPs al-ready us-ing CHI-CA | Total of 126 par-ticipants control group si-ze n.e.m.i.t. | Inter-vention 56%, control group: 62% | N.a. | N.a. | Self-reported use of validated screening tools for ASD. No changes in know-ledge or attitudes | N.a. |
| **Downs (2019)** | To determine whether com-puter-auto-mated scree-ning and clinical de-cision support can improve Autism Spectrum Disorder (ASD) scree-ning rates in pediatric pri-mary practi-ces | Cluster rando-mized clinical trial, com-paring ASD-screening rates in samples with or without screening module built into an existing decision support software system | Clinician’s res-ponse rates to screening results in the computer sys-tem | Screening rates, rates of positive screening results, new cases of ASD identified | The cluster randomized clinical trial was conducted between November 16, 2010 and November 21, 2012, outcomes were measured per patient | Decision sup-port with the CHICA, integrated with work-flow and with the electronic health record | N.e.m.i.t., ‘control clinics’ and ‘without an ASD screening module built into an existing decision support software system’ | Children aged 18 to 24 months in urban pediatric clinics of an inner-city county hospital system | 274 chil-dren | N.e.m.i.t. | 38.4% | N.e.m.i. t., age range 23-30 months | Among the 265 patients with positive screening results, physicians indicated any response in CHICA in 151 (57.0%) | Scree-ning rates in the inter-vention clinics (not in the con-trol clin-ics) in-creased from 0% at base-line to 68.4% in 6 months and to 100% in 24 months. Scree-ning results were positive for 265 of 980 children screened |
| **Carrol (2013)** | To determine if implement-ting Atten-tion-De-ficit/Hy-peractivity Disorder (ADHD) diagnosis and treatment gui-delines in a CDSS would result in better care, inclu-ding higher rates of adhe-rence to clinical care guidelines | A cluster rando-mized controlled trial in which the diagnosis and mana-gement of ADHD was stu-died af-ter imple-mentation of a CDSS in 4 pra-ctices. In the control group, screening was left to the dis-cretion of the phy-sician | Rate of use of structured diagnostic assessments | Number of ADHD core symptoms noted at time of diagnosis. Medication adjustments, reassessment of symptoms, mental health referral and visits | Data collection began 6 months after the mo-dule turned on in CHICA, continued until 6 months after the last patient was diagnosed with ADHD | CHICA ADHD-module | The ‘tra-ditional’ CHICA system  without the ADHD guidelines | Children with symp- toms or signs of ADHD | A total of 84 pa-tients. In the inter-vention and in the con-trol gro-up 42 pa-tients | N.a. | In inter-vention group 31%, in control group 26% | N.e.m. i.t. (age range 5-12 ye-ars) | The rate of use of structured diagnostic assessments incea-sed significantly | The num-ber of ADHD core symp-toms noted at time of diagnosis vastly in-creased, prelimi-nary ana-lyses showed an in-crease in ADHD manage-ment |
| **Fortney (2010)** | To describe the develop-ment and functionality of a decision support system for the chronic care model of depression treatment, known as collaborative care | Observa-tional study on a joint develop-ment of the program by a cross-functional design team of psychia-trists, depression care manager, informa-tion technology specialists, technical writers and researchers | Functional capabilities of NetDSS: clinical decision support, progress note generator, and workload and outcomes report generator | Number of patients being subjected to the NetDSS. Functional capabilities of NetDSS: patient registry, patient encounter scheduler, trial management | N.a. | Net- DSS | N.a. | N.e.m.i.t. (‘patients’, ‘chronic’, ‘depres-sion’) | N.e.m.i.t. (‘three collabo-rative care imple-menta-tion research projects’ ‘invol-ving 11 DCMs and 845 patients’) | N.e. m.i.t. | N.e. m.i.t. | N.e. m.i.t. | Intervention protocols can be successfully converted to Web-based decision support systems that facilitate the implementation of evidence-based chronic care models into routine care with high fidelity | The NetDSS has been used to provide evidence-based de-pression care ma-nagement to more than 1700 primary care pa-tients. See Re-sults  (pro-vider) |
| **Goodman (2000)** | To describe and validate the DAWBA based on community and psychia-tric clinic samples | Observa-tional study on DAWBA diagnoses, indepen-dent correlates and SDQ-profiles (emotional, conduct-oppositio-nal and ADHD -hyper-kinetic disorders) | Outcomes related to DAWBA as an epidemiological measure (i.e. reliability and validity), mental health & service provision | See ‘Outomes (provider)’ | At baseline and after 4-6 mon-ths.follow-up | DAWBA | N.a. | Children and adole-scents of 5-16 years old | A total of 530 par-ticipants, 491 in commu-nity and 39 in clinical sample | N.a. | 49% in comm-unity and 21% in clinical sample | Com-munity sample 9.9 ye-ars, clinical sample 11.0 years | The rates of all psychiatric disorders were higher in the clinic than in the community sample. Subjects with and without DAWBA diagnoses differed markedly in external characteristics and prognosis. In the clinical sample, there was considerable overlap between DA-WBA and case note diagnoses | See Results (pro-vider) |
| **Ford (2013)** | To explore the application of the DAWBA as an adjunct to clinical practice with children in the UK | Rando-mized controlled trial of the disclosure of the DA-WBA to the as-sessing practitio-ner versus assessment at normal and ana-lyzed by ‘intention to dis-close’ | Outcomes related to parents and the child’s teacher, who filled in the SDQ; as part of the DAWBA | Probability diagnoses of psychiatric disorder(s). Level of functioning for the child was measured using the Children’s Global Assess-ment Scale (CGAS) | An initial as-sessment at baseline, and a second after 6 months follow-up | DAWBA- disclosed as-sessment | Assess-ment at normal | Patients susceptible for emo-tional, be-havioural, autism spectrum, attention deficit hy-peractivi-ty, eating, feeding and tic disorders. Attach-ment issues | In total 235 par-ticipants. In inter-vention group 117, in control group 118 | N.e. m.i.t. | N.e. m.i.t. | Children of 5-10 years | Exposure to the DAWBA increased agreement between the DAWBA and practitioners about anxiety disorders, but detected no other statistically signi-ficant increased agreement for other disorders, nor a reduced need for further assessment, the number of difficulties recog-nized or influence on outcomes | See Results (provi-der) |
| **McEwen (2016)** | To test the DAWBA as a tool for diag-nosing Autism Spectrum Disorder (ASD) in community mental health settings | A general pop-ulation sample was screened with the Childhood Autism Spectrum Test (CAST) (low score <12, high score ≥ 15). Parents filled in the ASD module of the DAW-BA, families were visited at home; also the Autism Diagnostic Interview–Revised (ADI-R) and the autism diagnostic observa-tion sche-dule (ADOS) were com-pleted | Sensitivity, specificity, PPV, NPV and the rate of correct classification by the tool | See Outcomes (provider) | Specific measurement moments unspecified, DAWBA interview on-line or by tele-phone, ADI-R and ADOS during home visits | DAWBA ASD-section | Children at risk of ASD | Children and ado-lescents presenting in commu-nity mental health set-tings, at  risk for ASD | A total of 377 par-ticipants, 101 un-affected co-twins of chil-dren with a diagno-sis of ASD, 164 adolesc-ents with low and 112 with high risk of ASD | N.a. | ASD-group  17%, co-twins group 47.5%, co-twins and low-risk group: 39% | ASD-group 10.0 years, affected co-twin group 9.9 years and unaffected co-twin group 11.6 years | DAWBA shows good test specifications: sensitivity (0.88) and specificity (0.85), high PPV (0.82-0.95) and NPV (0.90). 86% of children were correctly classified.  Improved per-formance when used in conjunction with ADOS. High ASD-score correlation with ADI-R | See Results (pro-vider) |
| **Moya (2005)** | To develop and validate the Eating Disorder (ED)-section of the DAWBA | Girls di-vided into three gro-ups were assessed with the ED-section of the DA-WBA | Sensitivity, specificity, predictive values and test-retest re-liability | See Outcomes (provider) | Two measure-ment moments. Baseline at the beginning of the study, re-test after 2-3 weeks | DAWBA ED-section | Clinical controls with de-pression, obsessive-compul-sive dis-order or gastro-intestinal disease; com-munity controls | Girls at risk for an ED | A total of 174 par-ticipants. 48 with an ED, 55 clini-cal con-trols in treatment for uni-polar depress-sion, ob-sesssive compul-sive dis-order or gastro-intestinal disease; 71 com-munity controls | N.a. | 100% | ED-group 16.0 years, clinical controls group 14.5 years, com-munity controls group 15.5 years | For the detection of any DSM-IV and ICD-10 ED, the final DAWBA diagnosis had a sensitivity of 100%, specificity of 94%, PPV of 88%, and a NPV of 100%. There was 95% agreement between the initial and repeat diagnoses (k = 0.81) | See Results (provi-der) |
| **Robinson (2018)** | To compare COMPASS to community clinician-choice treatment for the recovery from an initial psychotic disorder-episode | Cluster rando-mized study | N.a. | Self-reported medication visits and prescriptions side effects, Adherence Estimator-scale.  Biological outcomes: Vital signs, blood glucose | Prescription data monthly. Outcomes (pa-tient) at 0, 3, 6, 12, 18 and 24 months | COMPASS (NAVIG-ATE) | Commu-nity cli-nician choice | Patients aged 15-40 years | Total of 414 parti-cipants, 233 in intervention, 181 in control group | N.e.m. i.t. | 27% | 23 years | More medication visits & antipsychotic prescriptions.  Fewer side effects. Less sedation and anticholinergic side effects. No effect on other side effects.  Less nonadherence beliefs. Less increase of BMI. No effects on other vital signs and cardiometabolic laboratory findings | See Results (provi-der) |
| **Reid (2013)** | To examine the *mobile-type* program in primary care, in particular the extent to which the *mobiletype* could provide clinical as-sistance, en-hance doctor-patient rap-port and lead to pathways to care | Referred and e-ligible pa-tients were randomly assigned to a group in which mood, stress and daily act-ivities were self-monitored or a group in which only daily activities were self-monitored. Monitoring data were collabora-tively re-viewed with their general practitio-ner | General practitioners assessed the program. Doctor-patient rapport was assessed using the General Practice Assessment Questionnaire – Communication and Enablement subscales and the Trust in Physician Scale. Pathway to care was mea-sured using the Party Project’s Exit Interview. Researchers were double blinded to group allocation | Perceived understanding of patient mental health, assistance in clinical decision-making about diagnosis and medication/re-ferral | Both groups self-monitored areas of func-tioning for 2 to 4 weeks | Mobiletype | Attention-compari-son | N.e.m.i.t. (‘mild or more mental health concerns’) | A total of 114 par-ticipants were in-cluded, inter-vention group 68 and at-tention-compari-son gro-up 46 | N.e.m. i.t. | 71.9% | 18.1 years | Mobiletype: im-proved general practitioners’ un-derstanding of functioning and clinical decision-making regarding medication/referral/ deciding about diagnosis; positive impact on communi-cation, no impact on general practitioner-patient rapport nor pathways to care | See Results (provi-der) |
| **Fletcher (2019 and 2021)** | To determine whether sys-tematic identi-fication of patients’ symptom severity using a Decision Support Tool in general practice and provision of tailored treat-ment recom-mendations is clinically and cost effective compared to usual care | Pragmatic stratified rando-mised controlled trial. Partici-pants were recruited and classi-fied into three prog-nostic gro-ups | N.a. | Psychological distress, mea-sured on the 10-item Kes-sler Psycho-logical Dis-tress Scale | Six months post rando-misation | Prognosis matched care | Usual care plus atten-tion con-trol | Adults aged 18-75 years re-porting de-pressive or anxiety symptoms or use of mental health me-dication | 1671 patients | N.a. | 72.5% | 15.0 years | N.a. | Prognosis-matched care was asso-ciated with greater reduction in psycho-logical distress than usual care plus attention control at 6 months. This re-duction was seen in the severe prognos-tic group, but not in the minimal/mild group. No serious adverse effects were recorded |
| **Parker (2020)** | To assess the feasibility and acceptability of delivering the Youth StepCare service in Australian general practices | A 12-week uncontrol-led trial in two gene-ral practi-ces in NSW, Australia. Symptoms were as-sessed using two question-naires for depressive and an-xiety sym-ptoms; feasibility and accep-tability u-sing a bat-tery of  question-naires | General prac-titioner (GP) satisfaction with the service | Self-reported symptoms of anxiety or de-pression | Per patient, between Au-gust 2018 and January 2019 | A web-based universal screening service deli-vered via a mobile tablet, Youth Step-Care | N.a. | Youth patients aged 14 to 17 years who visi-ted a parti-cipating GP during the scree-ning pe-riod | Five GPs and 6 practice staff. Of 46 youth patients, 28 con-sented and 19 comple-ted the screening instru-ment | N.e.m. i.t. | 68.4% | 15.21 years | GPs and practice staff were satisfied with the service, reporting that there was a need for the service and that they would use it again | Nine reported sym-ptoms of anxiety or de-pression, two of which were new cases |
| **Kaye (2017)** | To describe a large collabo-rative care program that covers most of New York | Observational study since 2010 and pre-post evaluation of training | Number of registrations, phone calls and face-to-face eva-luations. Two-weekly self-reported sa-tisfaction,  pre-post training knowledge, skills, confidence | N.a. | PCP satisfac-tion 2-weekly after phone consultation. Perceived knowledge, skill and confidence anually 2013-2015 | CAP PC | N.a. | PCPs | 1931 regi-stered PCPs | N.e. m.i.t. | N.e. m.i.t. | 12.5 ye-ars | CAP PC has provided 8013 phone consul-tations and 17523 CME credits over 6 years. PCPs report very high levels of satisfaction and growth in confidence | N.a., practice level and patient level data are avail-able when there is enough funding for future research |
| **Gadomski (2014)** | To describe how project TEACH engages PCPs, lead to changes in practice and what factors influence sustainability | Semi-structured interviews among two groups of PCPs, trai-ned and untrained | Participation motivation, self-reported confidence, impact on clinical out-comes, such as medication prescription and developing treat-ment plans | PCP perceived impact on patient outcomes, such as aversion of bad outcomes, more effective detection of problems | Summer and fall of 2012 | Project TE-ACH, study focusses on CAP PC | N.a. | PCPs | Total of 40 parti-cipants. 30 trai-ned and 10 un-trained PCPs | 62% | N.a. | N.a. | Increased confidence and collaborative treatment in primary care. Sustainability depends on PCP practice context and implementation support | See Results (provi-der) |
| **Kerker (2015)** | To describe the impact of Project TEACH on the identi-fication and treatment of mental health conditions | Observa-tional study on trained (pre- to post-compari-son) and untrained PCPs | N.a. | Prescription practices, diagnoses and follow-up care | Pre- and post-training: after 0 and 6 months and 27 hours | CAP PC | Random sample of PCPs | Project TEACH-trained PCPs | A total of 376 parti-cipants, 176 in interven-tion, 200 in control group | N.e.m. i.t. | N.e.m. i.t. | N.e. m.i.t. (age range 0-21) | N.a. | More psycho-tropic medi-cation pre-scription in the trained group. Less (un-confir-med) effect on depress-sion diag-noses, medica-tion use and fol-low-up care |
| **Yellowlees (2008)** | To examine the diagnostic characteristics and referral outcomes for eMental Health from 10 primary care clinics in California | Analysis of 139 previous referrals of children via video-conferen-cing | N.a. | Rate of diagnosis of psychiatric disorders such as anxiety, cognitive decline, depression and psychosis | Per patient, ini-tially and at 3 months follow-up | eMH | N.a. | Children and adole-scents younger than 18 years old | 139 partici-pants | N.a. | N.e.m. i.t. | 10.7 years | N.a. | Improved assess-ment of psychia-tric disor-ders, esp- ecially attention deficit and mood disorders Video-conferen-cing improved mental health |
| **Epstein (2007)** | To test if a collaborative consultative service model would im-prove patient outcomes | Paediatric practices were as-signed to a group (not) receiving access to the service | Use of evidence-based practices by paediatricians, knowledge related to the use of titration trials | Children’s Attention Deficit/Hyperactivity Disorder (ADHD) symptom-matology by systematic monitoring of medication effectiveness and by use of the Conners Parent and Teacher Rating scales | Self-reported provider outcomes pre- and post-intervention. Child outcome measures 0, 3 and 12 months after start of the trial | Titration trials in the context of collaborative consultation treatment services | Periodic medi-cation main-tenance | Children with ADHD-related symptoms | Fifty two pedia-tricians and their 377 pati-ents | N.e.m. i.t. (‘the two groups did not differ in sex com-posi-tion’) | 36.3% | 7.8 years | Increased use of evidence-based practices. However, many paediatricians did not fully use the services | Reduc-tion in core ADHD-symp-toms |
| **Williams (2006)** | To evaluate General Practitioner (GP) satis-faction and outcomes of a consultation-liaison ser-vice provided by psy-chiatrists | Evaluation of tele-phone advice and one-off assessment with feed-back if ne-cessary | GP and psychiatrist satisfaction & perceived barriers | N.a. | N.e.m.i.t. | Consultation-liaison | N.a. | GPs & psychiatri-sts | 167 GPs and 27 psychia-trists | N.e.m. i.t. | N.e.m. i.t. | N.e.m.i. t. | High GP and psychiatrist satisfaction. Increase in GPs’ knowledge and confidence while managing mental health problems. 100% retention of psychiatrists throughout the project | N.a. |
| **Jacob (2012)** | To establish a telepsychiatry consultation practice for children in rural areas | A 2-session telepsy-chiatry consulta-tion, con-sisting of a psychiatric evaluation session and a recom-mendation session | PCP satisfaction | Parental satisfaction | N.e.m.i.t. regarding satisfaction. Child Behavior Checklists (CBCLs) at 0, 3 and 6 months | Telepsychia-try consulta-tion practice | N.a. | PCPs | 15 chil-dren | N.e.m. i.t. | 33% | 9 years | Satisfaction as reported by PCPs was high | Parental satisfac-tion was high. Not enough follow-up CBCLs were re-turned to determi-ne cha-nges in patient symp-toms |
| **Walter (2019)** | To assess the structure and process of pediatric behavioural health integration and outcomes in patient experiences (access and quality), cost and provider satisfaction | Evaluation of a multi-compo-nent, trans-diagnostic integrated behaviour-ral health model in a large pe-diatric primary care net-work in Massa-chusetts, launched in 2013 | Practice-level behavioural health integration, ambulatory behavioural health spending, self-efficacy and professional satisfaction from participation | Practice-level psychotherapy, medical behavioural health visits and guideline-congruent medication prescriptions | At baseline and 5-year follow-up | Behavioral Health Integration Program | Care as usual (n.e.m.i.t.) | Pediatric primary care prac-titioners | ~105 PCPs serving ~114000 patients | N.e. m.i.t. | N.e. m.i.t. | N.e.m.i.t. | Increased practice-level behavioral health integration, total ambulatory behavioral health spending increased by 8% in constant dollars over 5 years, mainly attributable to task-shifting from specialty to primary care. Total emergency behavioral health spending decreased by 19%. Providers reported high behavioral health self-efficacy and professional satisfaction from participation | Increased practice-level psy-chothe-rapy and medical behavio-ral health visits, guideline con-gruent medica-tion pre-scriptions for anx-iety, de-pression and ADHD |
| **Malas (2019)** | To obtain qualitative and quan-titative infor-mation from PCPs relating to their expe-rience in using tele-phonic con-sultation ser-vices with child and adolescent psychiatrists through the Michigan Collaborative Child Care Program | A survey was con-ducted o-ver a 5-year period to assess PCP at-titudes and perception regarding MC3 con-sultation, including measures of effi-ciency, user-friendli-ness and confidence in provi-ding men-tal health-care | See ‘methods’ | N.a. | Over 5 years, following each consultation | Michigan Child Col-laborative Care (MC3) Program | N.a. | PCPs | 1241 PCPs enrolled in MC3 (44% response rate) | N.e.m. i.t. | N.e.m. i.t. | N.e.m.i.t. | Common themes elicited included perception of improved patient care, improved comfort and confidence, greater comfort with prescribing and monitoring of psychotropics and improved access to mental healthcare | N.a. |
| **Thomp-son (2019)** | To analyze effectiveness of screening, referrals and treatment uptake of a collaborative care for depression intervention across 10 primary care clinics in Chicago | Patients were screened with the Patient Health Qu-estionnaire-2 and -9. Electronic health re-cord data were ana-lyzed | N.a. | Depression symptoms. Sample cha-racteristics, screening ra-tes, referrals and treatment pathways | Between November 2016 and December 2017 | Collaborative Care for Depression of Adults and Adolescents | N.a. | Adults and Adole-scents with symptoms indicative of Major Depressive Disorder | 1008 patients | N.a. | 63.0% | N.e.m. i.t., 20% of the sample were between ages 12 and 25, and 19% were between ages 26 and 35 | N.a. | Scree-nings, referrals and uptake occurred propor-tionately across subgro-ups except for pa-tients a-ges 12-17. Ado-lescent age was associate with dis-propor-tionate Patient Health Quest-ionnaire-9 scree-nings and with tre-atment disenga-gement |
| **Campbell (2021)** | A quality improvement study to 1) increase the proportion of visits with screening for autism and 2) to increase the proportion of visits with referrals for autism eva-luation | Process changes were im-plemented in 3 pha-ses: 1) changing screening instrument and adding decision support 2) adding automatic reminders & 3) adding a referral option for autism evaluations | N.a. | Proportion of visits with autism scree-ning at 2 inter-vention clinics before and after imple-mentation of process chan-ges versus 27 community clinics | During primary care visits, over 2 years (base-line and phased improvements) | Process changes of universal screening for autism | Care as usual, n.e.m.i.t. (‘commu-nity cli-nics’) | Children susceptible for autism | 12233 well-child visits | N.a. | 48.0% in in-terven-tion and 48.8% in com-munity sample | 45.3% <24 ye-ars in in-terven-tion, 55.0% in com-munity sample | N.a. | Improved autism screening and refer-rals. Au-tism screening increased by 52% in inter-vention, 21% in commu-nity clinics. See study for results per phase |
| Legend CDSM = Clinical Decision-Support Method PCP = Primary Care Physician N.a. = Not Applicable N.e.m.i.t. = Not Explicitly Mentioned in Text | | | | | | | | | | | | | | |
